# Exploring motor imagery as a therapeutic intervention for Parkinson’s disease patients: a scoping review

**DOI:** 10.3389/fneur.2024.1422672

**Published:** 2024-11-01

**Authors:** Maxime Michel, Elena Terragno, Matthieu Bereau, Eloi Magnin, Nicolas Gueugneau, Antonio Vinicius Soares, Yoshimasa Sagawa

**Affiliations:** ^1^Department of Rehabilitation Science, University of Franche-Comte, Besançon, France; ^2^Integrative and Clinical Neurosciences UMR 1322 INSERM, University of Franche-Comte, Besançon, France; ^3^ERCOS Group, ELLIADD Laboratory EA4661, UTBM University of Franche-Comte University, Besançon, France; ^4^Postgraduate Programme in Health and Environment - University of Joinville Region, Joinville, Brazil

**Keywords:** Parkinson’s disease, motor imagery therapy, mental practice, neurorehabilitation, rehabilitation

## Abstract

**Background:**

Motor imagery (MI) has emerged as a promising therapeutic approach for Parkinson’s disease (PD). MI entails mentally rehearsing motor actions without executing them. This cognitive process has garnered attention due to its potential benefits in aiding motor function recovery in patients. The purpose of this review was to highlight the findings observed in motor symptoms, balance, gait, and quality of life.

**Methods:**

A literature search was carried out in Medline, Embase, Cochrane, and Physiotherapy Evidence Database (PEDro), from the first publication to February 2024. Studies with at least one keyword to PD and MI in the title were included.

**Results:**

The analysis included 53 studies out of the 262 identified. These comprised 12 randomized controlled trials (RCTs) with an average PEDro score of 6.6 out of 10, as well as 41 non-RCT studies. Notably, the majority of the RCTs focused on balance, gait, and lower limb exercises. The experimental group found an 85.2% improvement on the Timed Up and Go (TUG) with a cognitive task (*p* < 0.02), 5.8% improvement on the TUG (*p* < 0.05), and 5.1% improvement in walking speed (*p* < 0.05). Other variables did not show significant improvement. In descriptive and non-RCT studies, there were various tasks and outcomes for the lower and upper limbs. It has been demonstrated that there was no difference in execution time in MI between patients and healthy subjects (HS), whereas motor execution was slower in patients. Several tasks were analyzed for the upper limb, including thumb opposition, joystick movements, and writing tasks with variable results. RCTs were more focused on balance, lower limbs, and walking. There was no specific outcome regarding the upper limb or speech. Additionally, the heterogeneity of tasks and outcomes across studies is also a limitation.

**Conclusion:**

Current research on walking disorders in PD shows promise, but further investigations are crucial, particularly with an emphasis on upper limb function and speech. Studies with larger sample sizes and more precise methodologies are needed to enhance our understanding of the potential benefits of MI within the framework of comprehensive PD rehabilitation.

## 1 Introduction

Parkinson’s disease (PD) is the second most common neurodegenerative disorder after Alzheimer’s disease and a major cause of disability among the elderly. Although advancing age is linked to a heightened risk of PD, it remains uncertain whether this increase follows a linear or exponential pattern. A recent study underscored the need for higher-quality epidemiological data to ensure equitable representation across race, ethnicity, geography, sex, and gender (1). PD is caused by the loss of dopaminergic neurons, resulting in both motor and non-motor symptoms (2, 3). In PD patients, there are four primary clinical aspects: bradykinesia or akinesia, resting tremor, rigidity, and postural instability (2–10) whereas the non-motor symptoms include sleep disorders, depression, and digestive disorders (11). PD impacts sensorimotor functions such as walking, balance, and posture, leading to a decrease in the patient’s independence and participation in societal activities (12).

Parkinson’s disease (PD) presents various treatment options, with pharmacological approaches being the most prevalent. These treatments primarily focus on dopamine and its derivatives to manage symptoms (4). Although levodopa is widely recognized as the most effective medication for treating motor symptoms, there exist other medications such as monoamine oxidase type B inhibitors, amantadine, anticholinergics, *β*-blockers, or dopamine agonists. Its utilization is conditioned by the symptoms exhibited by the patient (13). Although this treatment is the most used, adverse effects such as dyskinesias and motor complications can be observed (14). This is one of the main reasons why other forms of symptomatic treatment have been researched. Among non-pharmacological treatments, physiotherapy has shown beneficial effects in the management of PD (5). Recent studies have shown positive effects on motor symptoms (5), quality of life (15), walking, and balance (5, 16, 17).

Among physiotherapy techniques, motor imagery (MI) was proposed more than 30 years ago as a potential tool of rehabilitation (18). It is defined as a mental process in which a person simulates a mental simulation of a motor act without making any movement (7, 8). This approach relies on the premise that MI and actual motor execution elicit activation in overlapping brain regions (19). Consequently, enhancing the engagement of motor regions in the brain (9) is a central objective of this technique.

MI, a recently developed approach for the rehabilitation of patients with PD, is supported and promoted for implementation in rehabilitation protocols as a promising approach (6, 20, 21). Several studies have demonstrated that combining MI with physiotherapy can be effective for patients with PD (6, 22). MI can be performed from a first-or third-person perspective (7, 23) and can be used for different modalities such as upper limb, lower limb, walking, and others. There are also numerous MI protocols based on distinct sensorimotor tasks (24–29), such as the goal-directed task and the Box and Block Test (BBT) (26), the MI of walking along a straight course (24), and the MI of walking forward, backward, and turning (25). Considering these different MI modalities, choosing the best MI protocol for a clinical application seems difficult, especially considering the procedures and possible expected benefits. Only one study has proposed a framework for motivational interviewing to help physiotherapists integrate MI into their clinical practice (27). In alignment with the imperative to optimize the clinical use of MI as a rehabilitation tool, this scoping review aimed to achieve two primary objectives. First, it was aimed to provide a comprehensive summary of the diverse MI protocols designed for patients with Parkinson’s disease (PD), to provide guidance and facilitate their application in clinical practice. Second, the review sought to highlight the key findings observed in these studies regarding motor symptoms, balance, gait, and quality of life.

## 2 Materials and methods

This review was conducted in accordance with the Preferred Reporting Items for Systematic Review and Meta-Analyses extension for Scoping Reviews (PRISMA-ScR) guidelines (Annex I). Based on our previous research, there is no existing scoping review on this subject.

### 2.1 Data sources and searches

Prospective research was carried out on four different databases, namely MEDLINE (PubMed), Embase, Cochrane (Cochrane library), and Physiotherapy Evidence Database (PEDro), from the initial publication until February 2024. To identify relevant articles, the following keywords and operators were used: “Parkinson disease”* OR “Parkinson Disease” OR “Parkinson’s disease”* AND “motor imagery”* OR “motor imagery practice”* OR “mental practice”*. In order to enhance the comprehensiveness of the potential articles included, the search was conducted using Medical Subject Headings (MeSH) terms and non-MeSH terms (identified by an asterisk).

### 2.2 Study selection

First, all articles with at least one keyword regarding PD and MI in the title were included in this phase. Duplicated articles were removed.

**Figure 1:**
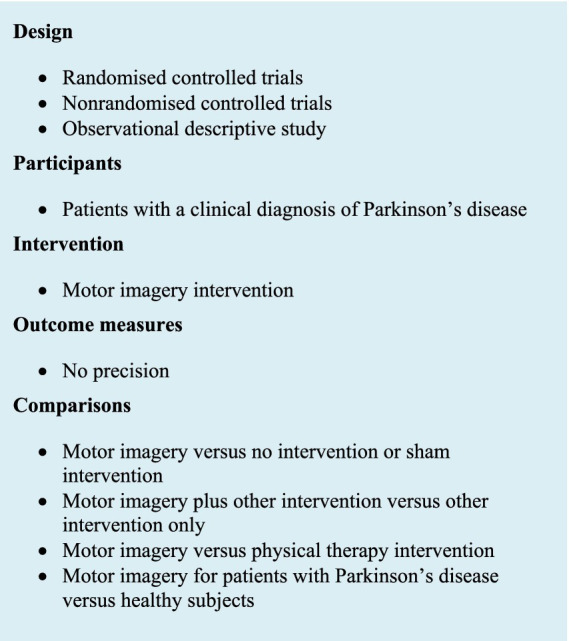
Eligibility criteria.

The eligibility criteria (Figure 1) for this phase of selection were applied to the title and abstract of the articles. Exclusion criteria were articles that were neither in English nor in French, feasibility and pilot studies, conference abstracts, and articles that did not focus on the specific effectiveness of MI. Full text was directly reviewed with eligibility criteria when the abstract did not provide sufficient information. Then, eligibility criteria were applied to the full text.

### 2.3 Data extraction and quality assessment

For this review, the articles were selected and read by two reviewers, MM and ET. Disagreements in this phase were resolved by consulting a third evaluator (YS).

The methodological quality of the randomized controlled studies (RCTs) was assessed with the PEDro scale. This is an 11-item scale. It is used to assess the external validity (criterion 1), internal validity (criterion 2–9), and interpretability of the findings (criterion 10 and 11) of a clinical trial or group comparison study. The PEDro scale is scored on a 10-point system, where 0 indicates very poor methodological quality and 10 signifies excellent methodological quality.

### 2.4 Data synthesis and analysis

Reviewers extracted the following key data from each article: the type of study, population characteristics, inclusion/exclusion criteria, intervention/protocol, variable of interest, and PEDro score. The mean (± Standard Deviation [SD]) values for all variables, *p* values, and modifications in percentage (comparisons among interventions and groups) were collected.

## 3 Results and comments

### 3.1 Selection of articles

**Figure 2:**
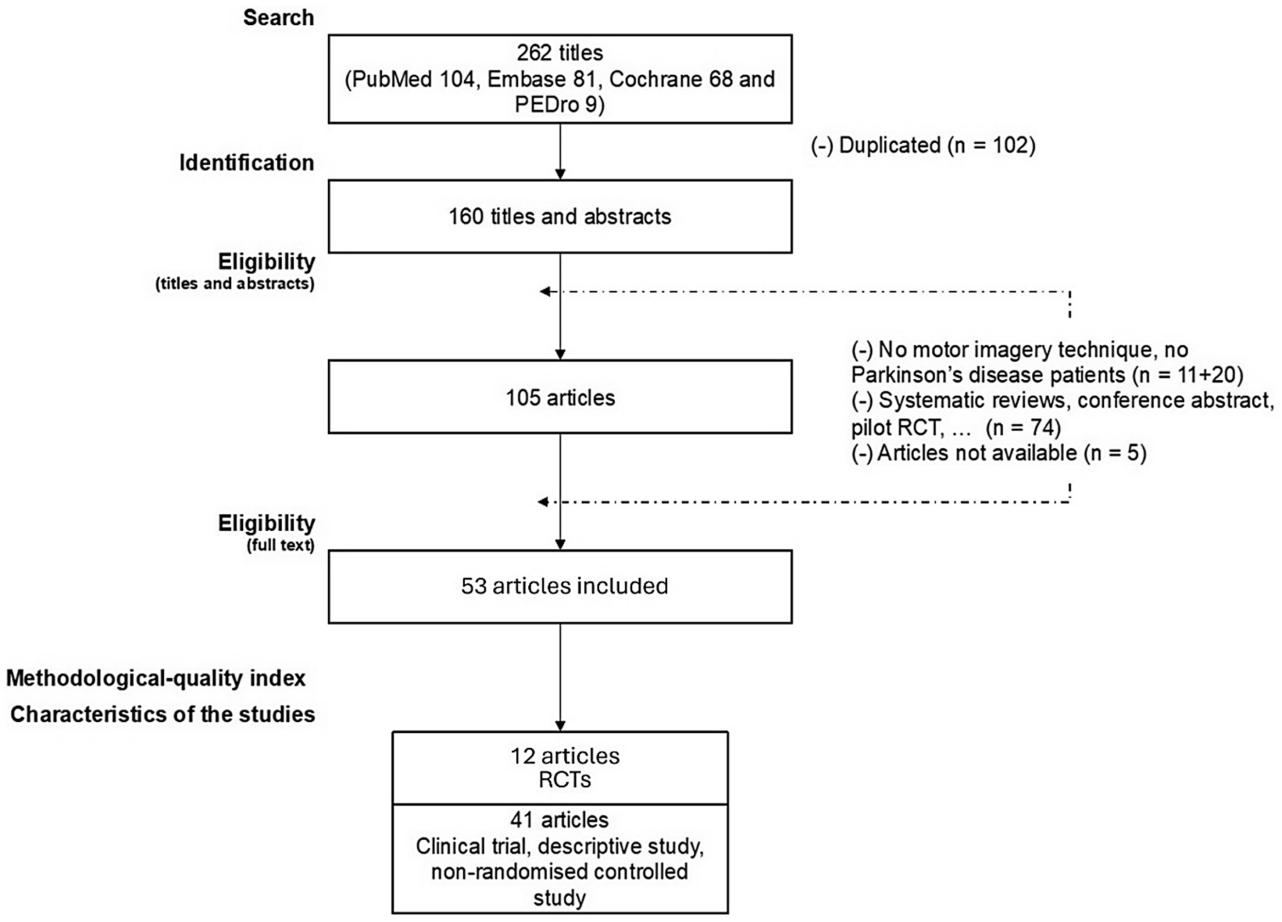
Flow of studies for the review.

Figure 2 shows the article selection process for this review. From the four databases combined, 262 articles were identified. A total of 53 articles were included, with 12 RCTs and 41 non-RCTs, as well as descriptive studies.

Methodological quality as assessed by the mean PEDro score for RCTs was 6.6/10, with only one being lower than 3/10 (30). All eligibility criteria, random allocation, baseline intragroup similarity, and between-group statistical comparison were respected for all studies. Although this was the case for the majority of RCTs, the blinding of participants and therapists was not consistently maintained.

### 3.2 RCT: effects of MI intervention

#### 3.2.1 Participants’ characteristics

The characteristics of RCTs are presented in Table 1. Participants’ characteristics were based on the diagnosis of PD. The mean (SD) number of participants per study was 29.9 (±10.5), with a mean age of 66.2 (±8.3) years. Groups were composed of an average of 30.7% of women and 69.3% of men. The mean (SD) Hoehn and Yahr (H&Y) score was 2.2 (0.5), with an off-phase score taken when it was specified.

**Table 1:** Characteristics of the randomized controlled trials.

Articles	Type of study	Participants: nb (nb per gender), mean (SD) age, mean (SD) UPDRS stage, mean (SD) H&Y score, treatment	Inclusion criteria (diagnosis, age, H&Y scale, MMSE score, others)	Exclusion criteria	Protocol (task, sessions [No. and W], frequency, intensity)	Evaluation (No., date, and outcomes)	PEDro score
Sarasso et al. (22)	RCT	Experimental group: 10 PD patients (5♀), 67.6 (6.4) y, H&Y OFF 5/4/3, UPDRS III 33.1 (11.9) Control group: 12 PD patients (5♀), 64.1 (8.9) y, H&Y OFF 5/4/4, UPDRS III 33.8 (10.5)	Idiopathic PD, H&Y score ≤ 3. Mini-mental score examination (MMSE) score (greater than or equal to 24)	Medical illnesses or substance abuse that could interfere with cognition; any (other) major systemic, psychiatric, neurological, visual, and musculoskeletal disturbances or other causes of walking inability; contraindications to undergoing MRI examination; and brain damage at routine MRI, including lacunae and extensive cerebrovascular disorders	Experimental group: Performed DUAL-TASK + AOT-MI (four gait/balance exercises each session were proposed with the following modality: 2 min of task observation → 5 min of task execution → 2 min of task imagination → 5 min of task execution) Control group: Performed DUAL-TASK the same number of exercises combined with watching landscape videos instead of observation/imagination For both groups: 1 h each session, 3 d/wk. for 6 wks	Primary clinical outcome: Kinesthetic and Visual Imagery Questionnaire (KVIQ) version 10, and brain MRI scans	7
Bezerra et al. (23)	RCT	Experimental group: 21 PD patients (7♀), 64.6 (9.3) y, H&Y OFF 2.0 (2.0–3.0), UPDRS II 23.0 (15.5–32.5), UPDRS III 13.0 (9.0–18.5) Control group: 18 PD patients (7♀), 60.7 (6.8) y, H&Y OFF 2.5 (2.0–3.0), UPDRS II 27.5 (18.0–41.2), UPDRS III 14.0 (10.0–23.0)	Idiopathic PD, H&Y scores 1.5 to 3; regular use of antiparkinsonian medication; walk independently for at least 10 meters without any orthosis or gait aid; no cognitive deficit according to the Mini-Mental state Examination (cutoff of 18 points for illiterate and 24 for those with school education)	Musculoskeletal or cardiorespiratory impairments affecting gait; and absence of other associated neurological diseases	Experimental group: Performed 12 sessions of AO, MI, and gait training. Control group: Watched PD-related educational videos and performed 12 sessions of gait training. For both groups: 1 h each session, 3 d/wk. for 4 wks	Primary clinical outcome: MiniBESTest: Mini Balance Evaluation Systems Test; FOG-Q: Freezing of Gait Questionnaire.	8
Kashif et al. (24)	RCT	Experimental group: 22 PD patients (9♀), 63.9 (4.6) y, H&Y OFF 2.1 (0.7), UPDRS II 22.0 (4.6), UPDRS III 32.5 (4.0) Control group: 22 PD patients (10♀), 2.3 (4.6) y, H&Y OFF 2.6 (0.7), UPDRS II 21.5 (3.9), UPDRS III 31.9 (4.6)	Idiopathic PD, severity ranging from stage I to stage III on the modified H and Y scale, intact cognition according to their mini-mental score examination (MMSE) score (greater than or equal to 24)	Other neurological presentation, orthopedic pathology, visual anomalies, cardiovascular issues, severe dyskinesia or “on–off” phases, a history of surgery for PD, a history of virtual games used for treatment in the last three months, and virtual game phobia	Experimental group: physiotherapy + virtual reality (Nintendo Wii) + motor imagery Control group: Physiotherapy 60 min/d For both groups: 3 d/wk., for 12 wks with follow-up to 16 wks	Primary clinical outcome: MDS-UPDRS part II and III Secondary clinical outcome: Balance confidence and balance	7
Tinaz et al. (33)	RCT	Experimental group: 22 PD patients (12♀), 66.2 (8.1) y, MDS-UPDRS III 32.3 (8.1), H&Y OFF 2.0 (0.2), NI Control group: 22 PD patients (12♀), 65.7 (8.8) y, MDS-UPDRS III 34.5 (9.6), H&Y OFF 2.1 (0.3), NI	Idiopathic Parkinson’s disease (according to UK Brain Bank criteria) Age ≥ 40 y Stable dopaminergic treatment during the study	H&Y scale >stage 3 Not fully independent Neurological or psychiatric disorder Medical condition that might affect central nervous system History of alcohol or illicit drug abuse Head injury resulting in loss of consciousness MoCA <21 Contraindications for MRI Poor homework compliance (<50%)	Experimental group: neurofeedback kinesthetic MI (walking, balance exercises, calisthenics) Control group: visual imagery exercises For both groups: 4 W, every day Tested in off-state	2 at W0 and after training Primary clinical outcome: MDS-UPDRS part III Secondary clinical outcome: 2 min endurance walking, TUG, 5 times sit-to-stand, 360-degree turning, physical performance test Primary imaging outcome: change in right insula-dmFC functional connectivity strength	5
Sarasso et al. (38)	RCT	Experimental group: 13 (5♀), 67.5 (6.1) y, MDS-UPDRS II 10.38 (5.55), H&Y ON 2.33 (0.5)/OFF 2.44 (0.5), NI Control group: 12 (4♀), 63.8 (9.2) y, MDS-UPDRS II 12.58 (5.14), H&Y ON 2.38 (0.5)/OFF 2.5 (0.5), NI	H&Y score ≤ 4 Postural instability and gait disorders phenotype Stable dopaminergic medication for at least 4 weeks, w/out any changes during observation period No dementia, MMSE ≥24 No significant head tremor	Medical illnesses or substance abuse that could interfere w/ cognition Other major systemic, psychiatric, neurological, visual, and musculoskeletal disturbances or other causes of walking inability Contraindications to undergoing MRI examination Brain damage at routine MRI, including lacunae and extensive cerebrovascular disorders	Experimental group: gait/balance training with dual task exercises added with AOT-MI therapy Control group: gait/balance training with dual task exercises and watching landscapes For both groups: 6 W, 3/W, 1 h Tested in on-state	3 at W0, W6 and W14 TUG with cognitive (primary outcome) TUG TUG with manual dual task MiniBESTest ABC scale 10MWT PDQ-39 NFoG-Q	7
Mahmoud et al. (32)	RCT	Experimental group: 15 (4♀), NI, levodopa medication Control group: 15 (5♀), NI, levodopa medication	Idiopathic Parkinsonism with cognitive dysfunctions (confirmed with RehaCom) Age: between 50 and 65 years Modified H&Y scale: stage 1–3 Male and female Disease duration from 3 to 5 years Taking levodopa medication	Other symptoms of Parkinsonism Modified H&Y scale: stage 4–5 Damaged eyesight who could not recognize objects on a computer screen	Experimental group: MI with cues, relaxation, and breathing exercises, sit to stand task and exercises in standing position and the control group task Control group: mental cognitive exercises including memory recall, math exercises, mental arithmetic, dual tasking For both groups: 6 W, 3/W, 1 h Tested in on-state	2 at pre-training and post-training Attention and concentration level (RehaCom assessment tool) Reaction time (RehaCom assessment tool) Figural memory level and missed pictures	3
Monteiro et al. (36)	RCT	Experimental group: 7 (0♀), 64 (7) y, UPDRS NS, H&Y OFF 2 (1), treatment NI Control group: 7 (2♀), 62 (12) y, UPDRS NS, H&Y OFF 2 (0.5), treatment NI Initially, 22 patients with PD received intervention, but there were 8 follow-up losses	Age between 45 and 72 years H&Y scale: stage 1–3 Both genders	Other neurological diseases Decompensated systemic diseases Reduced cognitive level Unable to perform MI during KVIQ-20	All patients before randomization: motor physiotherapy Experimental group: MI practice of a step and home exercises with handbook Control group: home exercises with handbook All patients before randomization: 15 sessions of 40 min, 2/W MI practice: 10 sessions of 5–10 min, 2/W Home exercises with handbook: 12 W, 3/W, 50 min Tested in on-state	3 at baseline (evaluation), after motor physiotherapy (reevaluation 1), after mental practice (reevaluation 2) TUG DGI FES-I Brazil	7
Subramanian et al. (37)	RCT	Experimental group 15 (1♀), 67 (9) y, MDS-UPDRS-MS 23.3 (9.4), H&Y 1.6 (0.6), levodopa, and equivalent medication Control group 15 (3♀), 63 (11) y, MDS-UPDRS-MS 26.7 (12.6), H&Y 1.7 (0.5), levodopa, and equivalent medication	Diagnosis of PD H&Y scale: stage 1–3 No dementia or significant comorbidity and fulfilled safety requirements for MRI	NI	Experimental group: Homework employing MI + supervised motor training with virtual reality gaming Control group: supervised motor training on a gaming device Experimental group: MI homework 4 W, 7/W, 10 min + supervised training 3/W, 25 min, and 6 W, 1/W, 10 min of MI homework + supervised training 1/W, 25 min Control group: 4 W, 3/W, 25 min, and 6 W, 1/W, 25 min	2 at W-1 and 1 week after intervention Primary outcome: off medication MDS-UPDRS-MS 3 at W0, W4, and W10 Secondary outcome: on medication MDS-UPDRS-MS MDS-UPDRS-motor aspects of daily living MDS-UPDRS-non-motor aspects of daily living MDS-UPDRS-summer score PDQ-39	6
Santiago et al. (34)	RCT	Experimental group: 10 (NS), 61.30 (9.95) y, UPDRS-III 27.60 (10.04), H&Y 2.75 (range: 2–3), pharmacological treatment Control group: 10 (NS), 61.40 (9.05) y, UPDRS-III 20.90 (14.85), H&Y 2.25 (range: 2–3), pharmacological treatment	Modified H&Y: stages 2–3 Taking antiparkinsonian medication Walking independently without any orthosis or gait-assistive device for at least 10 meters Not having undergone stereotaxic surgery	NI	Experimental group: 1 session of MI + physiotherapy gait protocol Control group: physiotherapy gait protocol	4 at baseline, 10 min, 1 day, and 7 days after training Primary outcomes: stride length, total stance time Secondary outcomes: hip ROM, velocity, TUG	8
Fayez and Elwishy (35)	RCT	Experimental group: 13 (NS), 72 (3.5) y, UPDRS NS, H&Y 2.2 (0.3), pharmacological treatment Control group: 13 (NS), 71 (4.2) y, UPDRS NS, H&Y 2.3 (0.3), pharmacological treatment	H&Y scale: stage 1.5–3 MMSE ≥26 Stable pharmacological treatment	Neuromuscular problems that affected their motor performance Vestibular dysfunction H&Y scale ≥ stage 4	Experimental group: physiotherapy + MI of gait Control group: physiotherapy + watching documentaries . Both groups: 4 W, 3/W Physiotherapy: calisthenic exercises (15–20 min), practice of specific functions for the lower and upper limbs (15–20 min), and relaxation exercises MI of gait and documentaries: 25–30 min	2 W0 and W4 Step length, Walking velocity Excursions in the sagittal plane of the ankle, knee, and hip joints FGA	7
Braun et al. (31)	RCT	Experimental group: 25 (8♀), 70 (8) y, UPDRS NI, H&Y (range: 1–4), NI Control group: 22 (7♀), 69 (8) y, UPDRS NI, H&Y (range: 1–4), NI	Clinically diagnosed adults with Parkinson’s disease Being able to engage in mental practice (clinical judgment of the treating therapist, support from family, MMSE score)	Other conditions, such as stroke Rheumatic diseases Dementia prior to the onset of Parkinson’s disease and sufficient to cause persistent premorbid disability	Experimental group: physiotherapy + MI of locomotor tasks adapted for each participant Control group: physiotherapy + relaxation (sham intervention) For both groups: 6 W Physiotherapy: 1 h or 2 times 30 min MI: 20 min or 2 times 10 min	3 at W0, W6, and W12 VAS for gait improvement (0 ‘poor’ and 10 ‘excellent’) TUG 10MWT	8
Tamir et al. (25)	RCT	Experimental group: 12 (4♀), 67.4 (9.7) y, UPDRS NI, H&Y 2.29 (0.4), pharmacological treatment Control group: 11 (4♀), 67.4 (9.1) y, UPDRS NI, H&Y 2.31 (0.4), pharmacological treatment	Community-dwelling individuals with PD H&Y scale: stages 1.5–3 MMSE ≥26 points	Presence of neuromuscular or skeletal comorbidities that affected their motor performance H&Y scale: stage 4 Ailments that prevented from making moderate physical efforts	Experimental group: physiotherapy + MI practice Control group: physiotherapy only For both groups: 12 W, 2/W, 1 h Physiotherapy: calisthenic exercises (15–20 min), practice of specific functions for the lower limb and upper limbs (15–20 min), relaxation exercises MI practice: integrated in physiotherapy, either preceded the motor task or followed it Tested in on-state	2 at 1 day before and at the end of the intervention TUG Standing up and lying down Turning in place, 360 deg. Tandem stance Functional reach test Shoulder tug UPDRS Clock drawing Stroop test	6

Abbreviations: ♀, woman/women; ♂, man/men; 10MWT, 10-Meter Walking Test; ABC, Activities Balance Confidence; AOT, Action observation therapy; DGI, Dynamix Gait Index; FES-I, Falls Efficacy Scale International; FGA, Functional Gait Assessment; H&Y, Hoehn and Yahr; HS, healthy subjects; KVIQ-20, Kinesthetic and Visual Imagery Questionnaire-20; MDS-UPDRS(-MS), Movement Disorder Society-Unified Parkinson Disease Rating Scale-(Motor Scale); MI, motor imagery; MiniBESTest, Mini Balance Evaluation Systems Test; MMSE, Mini Mental State Examination; MoCA, Montreal Cognitive Assessment; NFoG-Q, New Freezing of Gait Questionnaire; NI, non-informed data; PD, Parkinson disease; PDQ-39, Parkinson’s Disease Questionnaire-39 items; SD, standard deviation; TUG, Timed Up and Go; W, week(s); Y, years; ROM, range of motion; VAS, visual analogue scale.

The majority of studies had as inclusion criteria a Hoehn and Yahr (H&Y) score ≤ 3 (23–25, 31–37), except for Sarasso et al. (38), who included patients with a H&Y score ≤ 4. One study failed to report eligibility criteria related to an H&Y score, and another study excluded patients with an H&Y score > 3 (31). For the exclusion criteria, in most studies, patients with neuromuscular, psychiatric, or neurological pathologies other than PD were excluded.

#### 3.2.2 Protocols

Regarding the 12 RCTs, the mean protocol duration was 7 weeks, ranging from a single session to 12 weeks with a mean number of sessions per week of 3 (range: 1–7). The duration of the interventions was determined for 7 studies, with a mean duration of 55 min for the experimental group (range: 35–80) and 52 min for the control group (range: 25–80). All studies performed a pre-intervention and post-intervention assessment, and 3 studies (28, 30, 32) included a follow-up intervention ranging from 1 week to 8 weeks after the end of the protocol. Regarding the types of exercises, eight studies (22–24, 28–30, 32, 38) used an MI protocol of gait and balance exercises or gait exercises only. One study (33) comprised a single-step protocol for MI.

#### 3.2.3 Outcomes

In terms of motor symptoms, two studies (32, 35) used the Movement Disorder Society’s (MDS) Unified Parkinson’s Disease Rating Scale (UPDRS) as the primary outcome. They compared Part III of UPDRS. Regarding the assessment of quality of life, only 4 studies (23, 24, 31, 35) assessed this parameter using the Parkinson’s Disease Questionnaire-39 (PDQ-39). The walking and balance abilities were assessed, including walking speed, step length, Timed Up and Go (TUG), Dynamic Gait Index (DGI), Functional Gait Assessment (FGA), 10-Meter Walk Test (10MWT), 2-min endurance walking test, sit-to-stand, or a balance test (23–25, 31, 33–36, 38). Six studies have focused on balance (23–25, 33, 34, 38). Lower limb range of motion (ROM) was also assessed in two studies, one (34) focusing on the hip and the other (35) evaluating the hip, knee, and ankle. No specific upper limb or speech outcomes have been assessed.

#### 3.2.4 Results of RCT

Among the 12 studies, there was a substantial range in the significance of intergroup differences. Out of these, 10 studies demonstrated a significant difference between groups after the intervention (Table 2).

**Table 2:** Results of randomized controlled trials.

Articles	Techniques used	Outcomes (pre-intervention comparison, post-intervention comparison)	*p*-value
Sarasso et al. (22)	Experimental group: DUAL-TASK + AOT-MI vs. Control group: DUAL-TASK	Experimental group: pre-intervention KVIQ 57.3 (19.5) vs.Control group: pre-intervention KVIQ 53.8 (24.8) Experimental group: pre-intervention brain MRI scans vs.Control group: pre-intervention brain MRI scans Experimental group: post-intervention KVIQ 70.0 (32.2) vs.Control group: post-intervention KVIQ 59.0 (34.9) Experimental group: post-intervention brain MRI scans vs.Control group: post-intervention brain MRI scans	N.S. N.A. *p* < 0.001 *p* < 0.001
Bezerra et al. (23)	Experimental group: AO, MI, and gait training vs. Control group: watched PD-related educational videos and gait training	Experimental group: pre-intervention MiniBESTest 24.2 (1.4) vs.Control group: pre-intervention MiniBESTest 23.6 (1.3) Experimental group: pre-intervention FOG-Q 9.3 (1.6) vs.Control group: pre-intervention FOG-Q 9.8 (1.5) Experimental group: post-intervention MiniBESTest 25.7 (1.4) vs.Control group: post-intervention MiniBESTest 24.2 (1.3) Experimental group: post-intervention FOG-Q 8.8 (1.6) vs.post-intervention FOG-Q 8.7 (1.5) Intergroup comparison difference * Only to MiniBESTest: domain—reactive postural control	N.S. N.S. *p* < 0.001 *p* < 0.001 * *p* = 0.01
Kashif et al. (24)	Experimental group: physiotherapy + virtual reality (Nintendo Wii) + motor imagery vs. Control group: Physiotherapy	Experimental group: pre-intervention UPDRS II 22.0 (4.6) vs.Control group: pre-intervention UPDRS II 21.5 (3.9) Experimental group: pre-intervention UPDRS III 32.5 (4.0) vs.Control group: pre-intervention UPDRS III 31.9 (4.6) Experimental group: pre-intervention Balance confidence—ABCS 59.6 (5.9) vs.Control group: pre-intervention Balance confidence—ABCS 59.3 (8.9) Experimental group: Balance—BBS pre-intervention 39.0 (3.2) vs. Control group: pre-intervention 40.2 (4.6) Experimental group: post-intervention UPDRS II 17.1 (4.4) vs.Control group: post-intervention UPDRS II 20.0 (3.8) Experimental group: post-intervention UPDRS III 23.0 (8.3) vs.post-intervention UPDRS III 28.2 (6.1) Experimental group: post-intervention Balance confidence—ABCS 59.6 (5.9) vs.Control group: post-intervention Balance confidence—ABCS 59.3 (8.9) Experimental group: Balance—BBS post-intervention 39.0 (3.2) vs. Control group: post-intervention 40.2 (4.6)	N.S. N.S. N.S. N.S. *p* < 0.001 *p* < 0.001 *p* < 0.001 *p* < 0.001
Tinaz et al. (33)	Experimental group: NF-guided kinesthetic MI vs. Control group: visual imagery training	MDS-UPDRS III, experimental group pre-intervention 32.3 (8.1) vs. control group pre-intervention 34.5 (9.6) Endurance walking, experimental group pre-intervention 162.6 (30.7) m vs. control group pre-intervention 152.7 (26.1) m Gross motor combined, experimental group pre-intervention 23.7 (4.7) s vs. control group pre-intervention 24.4 (4.9) s Physical performance test, experimental group pre-intervention 25.1 (3.3) vs. control group pre-intervention 24.2 (3.0) MDS-UPDRS III, experimental group post-intervention 31.3 (9.8) vs.control group post-intervention 35.1 (10.8) Endurance walking, experimental group post-intervention 171.3 (33.2) m vs. control group post-intervention 160.7 (25.5) m Gross motor combined, experimental group post-intervention 22.3 (5.1) s vs. control group post-intervention 24.1 (5.2) s Physical performance test, experimental group post-intervention 26.1 (3.5) vs. control group post-intervention 24.7 (3.5)	N.S. N.S. N.S. N.S. N.S. N.S. N.S. N.S.
Sarasso et al. (38)	Experimental group: dual-task+ AOT-MI vs. Control group: dual task only	TUG-COG delta W0-W6, experimental group-8.17 (12.75) s vs. control group-3.68 (7.18) s TUG delta W0-W6, experimental group-2.11 (1.69) s vs. control group-2.08 (2.64) s TUG-MAN delta W0-W6, experimental group-2.11 (2.61) s vs. control group-3.42 (6.67) s MiniBESTest delta W0-W6, experimental group 2.92 (2.02) vs. control group 0.33 (2.53) ABC scale delta W0-W6, experimental group 11.43 (9.11) vs. control group 2.53 (8.78) 10MWT-confortable speed delta W0-W6, experimental group-1.01 (1.11) vs. control group-0.18 (0.97) PDQ-39 delta W0-W6, experimental group-4.61 (5.70) vs. control group-0.62 (8.44) TUG-COG delta W0-W14, experimental group-6.29 (9.94) s vs. control group-4.24 (7.94) s TUG delta W0-W14, experimental group-2.04 (1.69) s vs. control group-2.82 (2.92) s TUG-MAN delta W0-W14, experimental group-1.70 (2.18) s vs. control group-3.45 (5.75) s MiniBESTest delta W0-W14, experimental group 3.27 (2.72) vs. control group 0.67 (3.55) ABC scale delta W0-W14, experimental group 11.53 (11.78) vs. control group 0.76 (9.76) 10MWT-confortable speed delta W0-W14, experimental group-1.65 (2.01) vs. control group-0.33 (0.73) PDQ-39 delta W0-W14, experimental group-4.14 (6.77) vs. control group-4.28 (5.72)	*p* < 0.001 *p* = 1.0 *p* = 0.21 *p* = 0.01 *p* = 0.01 *p* = 0.05 *p* = 0.38 *p* = 0.02 *p* = 1.0 *p* = 0.15 *p* = 0.02 *p* = 0.03 *p* < 0.001 *p* = 0.41
Mahmoud et al. (32)	Experimental group: MI with augmented cues + specifically designed intervention vs. Control group: specifically designed intervention	Attention and concentration level: experimental group pre-7.46 vs. control group pre-7.8 Reaction time in attention and concentration: experimental group pre-9096.4 ms vs. control group pre-9178.46 ms Figural memory level: experimental group pre-5.53 vs. control group pre-5.06 Missed pictures for figural memory: experimental group pre-9.06 vs. control group pre-8.86 Attention and concentration level: experimental group post-17.06 vs. control group post-10 Reaction time in attention and concentration: experimental group post-3085.06 ms vs. control group post-6949 ms Figural memory level: experimental group post-10 vs. control group post-7.06 Missed pictures for figural memory: experimental group post-2.13 vs. control group post-6.13	*p* = 0.55 *p* = 0.90 *p* = 0.46 *p* = 0.83 *p* < 0.001 *p* < 0.001 *p* <0.001 *p* < 0.001
Monteiro et al. (36)	Experimental group: MI + home exercise guidelines handbook vs. Control group: handbook activities only	TUG evaluation, experimental group vs. control group, no data available DGI evaluation, experimental group vs. control group, no data available FES-I evaluation, experimental group vs. control group, no data available TUG reevaluation 1, experimental group vs. control group, no data available DGI reevaluation 1, experimental group vs. control group, no data available FES-I reevaluation 1, experimental group vs. control group, no data available TUG reevaluation 2, experimental group vs. control group, no data available DGI reevaluation 2, experimental group vs. control group, no data available FES-I reevaluation 2, experimental group vs. control group, no data available	N.S. N.S. N.S. N.S. N.S. N.S. *p* = 0.05 N.S. N.S.
Subramanian et al. (37)	Experimental group: homework employing MI + motor training with a virtual reality vs. Control group: motor training with a virtual reality gaming device	Primary outcome (off medication) MDS-UPDRS-MS, experimental group pre-post-4.5 (3.3) vs. control group pre-post-1.8 (8.3) Secondary outcome (on medication) MDS-UPDRS-MS, experimental group pre-post-4.9 (3.8) vs. control group pre-post-5.4 (4.9) MDS-UPDRS-M-DL, experimental group pre-post-1.7 (2.3) vs. control group pre-post-1.5 (2.8) MDS-UPDRS-NM-DL, experimental group pre-post-2.8 (2.9) vs. control group pre-post-0.9 (3.9) MDS-UPDRS-SS, experimental group pre-post-9.2 (9.7) vs. control group pre-post-7.9 (8.4) PDQ-39, experimental group pre-post-2.4 (4.8) vs. control group pre-post-3.6 (6.5)	*p* = 0.73 *p* = 0.86 *p* = 0.86 *p* = 0.73 *p* = 0.86 *p* = 0.93
Santiago et al. (34)	Experimental group: MI added to physiotherapy vs. Control group: physiotherapy	Stride length: experimental group pre-11.1 (0.1) m vs. control group pre-1.17 (0.1) m Total stance time: experimental group pre-1.37 (0.06) s vs. control group pre-1.47 (0.06) s Hip ROM: experimental group pre-33.9° (1.6) vs. control group pre-36.6° (1.6) Velocity: experimental group pre-1.05 (0.06) m/s vs. control group pre-1.06 (0.06) m/s TUG: experimental group pre-12.6 (1.0) vs. control group pre-13.1 (1.2) Stride length: experimental group post-1.17 (0.05) m vs. control group post-1.18 (0.05) m Total stance time: experimental group post-1.34 (0.06) s vs. control group post-1.45 (0.06) s Hip ROM: experimental group post-36.1° (1.7) vs. control group post-38.2° (1.7) Velocity: experimental group post-1.12 (0.07) m/s vs. control group post-1.09 (0.7) m/s TUG: experimental group post-11.3 (0.8) vs. control group post-12.0 (0.9)	*p* < 0.05 N.S. *p* < 0.05 *p* < 0.05 *p* < 0.05
Fayez and Elwishy (35)	Experimental group: MI of gait + physiotherapy vs. Control group: physiotherapy	Speed: experimental group pre-0.74 (0.02) m/s vs. control group pre-0.75 (0.03) m/s Step length: experimental group pre-0.50 (0.07) m vs. control group pre-0.51 (0.05) m Hip ROM: experimental group pre-39.5° (6) vs. control group pre-39.3° (5.7) Knee ROM: experimental group pre-45.7° (7.1) vs. control group pre-47.7° (5.4) Ankle ROM: experimental group pre-19.2° (5.5) vs. control group pre-20.4° (4.8) FGA: experimental group pre-15.5 (3) vs. control group pre-16.2 (2.8) Speed: experimental group post-0.87 (0.02) m/s vs. control group post-0.81 (0.03) m/s Step length: experimental group post-0.60 (0.03) m vs. control group post-0.55 (0.05) m Hip ROM: experimental group post-54.7° (7.2) vs. control group post-48.1° (6.1) Knee ROM: experimental group post-60.7° (9.3) vs. control group post-52.5° (6) Ankle ROM: experimental group post-29.2° (5.4) vs. control group post-24.8° (4.6) FGA: experimental group post-21.8 (3.2) vs. control group post-18.8 (2.8)	*p* = 0.61 *p* = 0.84 *p* = 0.92 *p* = 0.44 *p* = 0.57 *p* = 0.55 *p* < 0.001 *p* < 0.001 *p* = 0.02 *p* = 0.01 *p* = 0.04 *p* = 0.02
Braun et al. (31)	Experimental group: physiotherapy + MI vs. Control group: physiotherapy + relaxation (used as a sham intervention)	VAS walking (participant rating): experimental group pre-5.0 (2.2) cm vs. control group pre-6.5 (2.1) cm TUG: experimental group pre-14.6 s (9.6) vs. control group pre-15.7 s (16.5) 10MWT: experimental group pre-10.3 s (3.6) vs. control group pre-11.0 s (5.1) VAS walking (participant rating): experimental group post-5.5 (2.1) cm vs. control group post-6.9 (1.7) cm TUG: experimental group post-18.1 s (31.6) vs. control group post-9.5 s (1.5) 10MWT: experimental group post-11.8 s (12.6) vs. control group post-8.3 s (1.5)	N.S. N.S. N.S. N.S. N.S. N.S.
Tamir et al. (25)	Experimental group: MI + physiotherapy vs. Control group: physiotherapy	Functional reach: experimental group post-vs.control group post, no data available UPDRS 1: experimental group pre-post-vs.control group pre-post, no data available UPDRS 2: experimental group pre-post-vs.control group pre-post, no data available UPDRS 3: experimental group pre-post-vs.control group pre-post, no data available UPDRS 6: experimental group pre-post-vs.control group pre-post, no data available Clock drawing: experimental group post-vs.control group post, no data available	N.S. N.S. N.S. N.S. N.S. N.S.

Regarding the studies with gait and balance MI exercises, Sarasso et al. (38) reported a significant improvement in TUG with a cognitive task (primary outcome) compared to the control group. An improvement of 122% (*p* < 0.001) was found in week 6, and 48.3% (*p* = 0.02) in week 14. Furthermore, Santiago et al. (34) found an improvement in the TUG for the experimental group (5.8%; *p* < 0.05). Sarasso et al. (38) demonstrated an improvement of 388.05% (*p* = 0.02) during week 14 for the experimental group for the Mini Balance Evaluation System Test, as well as an improvement of 1417.1% (*p* = 0.03) for the Activities-specific Balance Confidence Scale. Mahmoud et al. (32) examined concentration parameters of motor learning. For the attention and concentration program, they used a questionnaire based on reaction time to identify matched figures. The motor learning test was based on a computer-based cognitive assessment device (RehaCom). The degree of attention and concentration was significantly improved by 70.6% (*p* < 0.001). The reaction time of the previous test was also improved by 55% (*p* < 0.001). Two other variables on figural memory were also improved (range: 42–65%; *p* < 0.001). Fayez and Elwishi (35) observed a significant difference in hip, knee, and ankle ROM in the experimental group (range: 13.7–17.7%; *p* < 0.01–0.04). For the spatiotemporal parameters, Fayez and Elwishi (35) showed a significant improvement in walking speed by 7.4% (*p* < 0.001), step length by 9.1% (*p* < 0.001), and FGA by 16% (*p* < 0.02) in the experimental group. Santiago et al. (34) observed a significant improvement in walking speed (2.8%; *p* < 0.05) in the experimental group. Sarasso et al. (38) reported an improvement of 400% at week 14 for the 10MWT. Monteiro et al. (36) studied MI with only one-step execution and found a significant difference for the TUG test at 14 weeks (difference not specified; *p* = 0.05).

Another noteworthy result was discovered by Sarasso et al. (38), wherein the MI was assessed using a Kinesthetic and Visual Imagery Questionnaire (KVIQ) and a MI functional magnetic resonance imaging (fMRI) task. During the fMRI, the participants, 25 PD patients and 23 healthy people, were asked to watch videos in the first-person perspective depicting gait/balance tasks and mentally simulate their execution. They demonstrated that action observation therapy and MI training (AOT-MI) in PD patients promoted functional plasticity in the brain areas involved in MI processes and gait/balance control (22).

There are no outcomes available for the upper limb or speech, as no specific outcomes were assessed.

### 3.3 Non-RCTs and descriptive studies: assessment of MI and main results

The results of the subsequent studies should be interpreted with the utmost caution, as we solely focused on their main results. We have organized the results according to this logic: first, the difference between patients with PD and healthy subjects (HS) in terms of MI (PD/HS-MI); second, the difference between patients with PD and HS in terms of ME (motor execution) (PD/HS-ME); and finally, the difference between ME and MI (MI/ME) for the same group of patients. The characteristics of the descriptive and non-RCT studies are shown in Table 3, and the main results are shown in Table 4.

**Table 3:** Characteristics of non-randomized controlled trials and descriptive studies.

Assessment	Type	Articles	Type of study	Participants: nb (nb per gender), mean (SD) age, mean (SD) UPDRS stage, mean (SD) H&Y score, treatment	Inclusion criteria	Exclusion criteria	Task	Evaluation
Clinical assessment	MI of walking	Cohen et al. (43)	Descriptive study	Experimental groups: PD-FOG: 11 (2♀), 68 (8) y, UPDRS 44.9 (15.1), H&Y 3.0 (0.8), NI PD-nonFOG: 13 (3♀), 67 (6) y, UPDRS 32.2 (7.6), H&Y 2.1 (0.5), NI Control group: 10 HS (0 ♀), 67 (7) y	NI	Dementia or other neurological diseases Vestibular disorders Musculoskeletal gait impairment Inability to stand and walk for 20 min	Passability experiment: Judged if they could get through a door without rotating their torso Imagery experiment: Part A: ME and MI of walking to a line behind a sliding door (repeated with several opening sizes of the sliding door) Part B: constant door opening, but subjects started at different distances from the door. The experiment was conducted in ME and MI Tested in an “off” state	Passability experiment: passability estimation (% of body width) Imagery experiment: execution time
		Ehgoetz Martens et al. (44)	Descriptive study	PD-nonFOG group: 15 (3♀), 71 (9.4) y, UPDRS-III 24.3 (7.3), H&Y NI, treatment NI PD-FOG group: 9 (0♀), 73 (4.2) y, UPDRS-III 30.9 (9.9), H&Y NI, treatment NI	NI	Visual disturbances impairing distance acuity (Snellen Eye Chart >20/50) Poor contrast sensitivity (Peli-Robson chart <18/42) Gait impairments preventing individuals from walking 10 m unassisted Modified MMSE <70/100 Spatial working memory impairments	Experiment 1: pointing judgment and walking judgment toward a target placed between 2.5 and 7 m and then removed Experiment 2: walking to a target located between 3 and 6 meters and MI of this test Tested in an “on” state	Experiment 1: magnitude of error Experiment 2: execution time
fMRI		Huang et al. (47)	Descriptive study	PD-nonFOG group: 14 (42.9%♀), 69.8 (7.8) y, UPDRS 37.9 (18.0), UPDRS-III 24.4 (14.1), H&Y 2.2 (0.5), treatment NI PD-FOG group: 20 (40%♀), 66.0 (6.2) y, UPDRS 51.3 (20.1), UPDRS-III 30.4 (15.2), H&Y 3.1 (0.7), treatment NI Control group: 15 HS (66.7%♀), 63.4 (7.0) y	NI	NI	Video-guided MI of turning and straight walking with and without freezing Patients watched the video and mentally imagined themselves performing the action currently played Tested in an “off” state	BOLD response
		Maidan et al. (48)	Descriptive study	Experimental group: 20 (6♀), 72.9 (1.6) y, UPDRS-III 29.8 (2.4), H&Y NI, dopaminergic treatment Control group: 20 HS (10♀), 69.7 (1.3) y	For all participants: Age > 60 years Able to walk 5 min unassisted Stable medication for the past month For patients with PD: Idiopathic PD (according to UK Brain Bank criteria) H&Y scale: stage 2–3 Taking antiparkinsonian medication	Psychiatric disorders MMSE <24 History of stroke, traumatic brain injury, or chronic neurological disorders Orthopedic disorders that may affect gait	(1) MI of walking on a clear virtual path presented (2) MI of walking on a virtual path displayed with obstacles (3) Plan a path on a map displayed in front of them, then MI of walking while navigating Control task: watching the same virtual scenes without MI of walking 45 s for each walking tasks, 4 times Tested in an “off” state	Neural brain activation
fMRI	MI of walking	Peterson et al. (28)	Descriptive study	Experimental group: 19 (8♀), 64.9 (7.6) y, UPDRS 31.2 (10), H&Y 2.34 (0.33), levodopa (3 PD without treatment) Control group: 20 HS (15♀), 66.6 (7.6) y	Idiopathic PD Averaged >3 on both the visual and kinesthetic components of KVIQ-20 Included regardless of freezing status	Lower-limb injuries Contraindications for an MRI Neurological problems other than PD or cognitive dysfunction	Following tasks in MI and ME: forward walking, backward walking, turning to the left, turning to the right, standing quietly Motor imaging tasks are performed in an fMRI	Execution time BOLD with a region of interest
		Snijders et al. (46)	Descriptive study	Experimental group: 24 (9♀) (12 FOG, 12 nonFOG), 60.2 (8.9) y, UPDRS-III FOG 34.6 (9.6)/nonFOG 28.6 (12.2), H&Y NI, dopaminergic medication Control group: 21 HS (9♀), 57 (9.1) y	NI	Marked resting tremor Vividness of MIQ score > 200	2 tasks: MI of gait and a matched visual imagery control task (imagine seeing a disc moving along a path) For both tasks, 2 widths (narrow and broad), 5 different distances (2, 4, 6, 8, 10 m) ME of walking along the path with 2 widths, 5 different distances 2 sessions of 25 min for the MI of gait and visual imagery task Tested in an “off” state (12 h without medication)	Execution time (imagery task) Gait data (step length, gait asymmetry) ROI analysis for fMRI
PET scan		Maillet et al. (45)	Descriptive study	Experimental group: 8 (4♀), 63.3 (6.3) y, UPDRS-III off 37.8 (8.7)/on 14.9 (5.7), H&Y 3.4 (0.5), dopaminergic treatment Control group: 8 HS (4♀), 62.9 (6.7) y	Gait score items in UPDRS-III improved by at least 1 point on compared to off KVIQ-k score ≥ 30/50	For all participants: MMSE <27/30 Frontal assessment battery score: < 14/18 For patients with PD: Mattis dementia rating scale score < 130/144 Orthopedic or psychiatric disorders Marked resting tremor Neurosurgery	Behavioral session: MI of walking (distance of 6 and 10 m on a line of 27 cm and 9 cm wide), MI of walking on this line, and visual imagery (imagine a blue puck moving on this line) PET session: MI of walking (distance of 6 m and 10 m on a line of 27 cm wide), visual imagery (imagine a blue puck moving on the 6 m*27 cm or 10 m*27 cm) and control task (press a button after a beep) Tested in an “on” and “off” state	Behavioral session: KVIQ score, execution time PET session: execution time, rCBF
		Weiss et al. (49)	Descriptive study	Experimental group: 10 (NI), UPDRS-III STN-DBS ON 14.7 (4.8) /STN-DBS OFF 39.1 (7.1), H&Y NI, STN-DBS treatment	NI	NI	Actual gait: 2 times walking during the 90s on a 15 m route, walking on an 8 m-long wallpaper for stride length Stance: 90s standing on a 40*40 cm square MI: imagine walking on a 15 m route 30s, 60s, 90s Imagery stance: imagine stance for the 90s PET scan: 3 times each 4 conditions (STN-DBS ON/OFF, imagery of walking/stance) Tested in an “off” state	MI of walking distance Walking distance Stride length Velocity PET activation with rCBF
Behavioral assessment	Thumb opposition	Avanzino et al. (51)	Descriptive study	Experimental group: 14 (6♀), 68.78 (8.71) y, UPDRS-III (range: 5–37), H&Y (range: 1–2.5), dopaminergic treatment Control group: 12 HS (5♀), 64.15 (10.88) y	Diagnosis of PD according to the UK Parkinson’s Disease Society Brain Bank criteria H&Y scale: stages 1–3 Stable dopaminergic medication regimen	History of any neurological disease other than PD Ongoing functional brain surgery treatment MMSE corrected score: < 24 Visual or hearing impairment Severe orthopedic problems of the upper limb	Sequential opposition of thumb to index, medium, ring, and little fingers Two tasks: (1) the execution task: tap in synchrony (SYNC) with a metronome cue, and when the tone stops, they have to continue performing the sequential opposition (CONT-EXE) (2) the MI task, which starts with a phase with the metronome, and then when the tone stops, participants were requested to imagine finger tapping at the same rhythm (CONT-MI) Each phase (with metronome and without) lasted 45 s, two blocks for each task Tested in an “on” state	Temporal error Interval reproduction accuracy index
PET scan	Thumb opposition	Cunnington et al. (52)	Descriptive study	Experimental group: 6 (2♀), 66.0 (7.5) y, UPDRS NI, H&Y (range: 3–4), pharmacological treatment Control group: 3 HS (1♀), 60.7 (3.8) y	NI	NI	Task: finger-to-thumb opposition movement at 1 Hz for 50 s 16 PET scans per subject (for PD patients, 8 were in an off-state and 8 were in an on-state) Each PET scan has 2 conditions: MI or rest	Relative rCBF
Electrode recording		Leiguarda et al. (53)	Descriptive study	Experimental group: 3 (NI), median: 50 (range: 15) y, UPDRS NI, H&Y 4, treatment NI	Idiopathic PD according to UKPDS Brain Bank criteria Severe motor fluctuations	NI	Task: thumb to index opposition, flexion/extension of all fingers simultaneously, flexion/extension of the elbow, flexion/extension of the ankle 3 conditions: rest (30 s), MI (30 s for each movement), and ME (30 s for each movement)	Firing rate of the globus pallidus internus (microelectrode recording)
EMG	Hand gripping	Kobelt et al. (41)	Descriptive study	Patients with PD: 5 (NI), 65.4 (6.0) y, UPDRS NI, H&Y NI, treatment NI Patients with stroke: 7 (NI), 53.7 (16.3) y Healthy participants: 10 (NI), 45.4 (15.4) y	For all participants: age > 18 years, male and female; be able to sit on a normal chair with eyes closed; be able to do grasping and arm lifting tasks alone; have given written consent For patients with PD: idiopathic PD, no deep brain stimulation treatment For HS: no neurological or psychological disorders	Additional neurological, psychological, or psychiatric disorders Severe cardiovascular and pulmonary diseases Severe pain Severe upper limb deformation of joints with arthritic origin Impairments in cognition and communication	Task: Hand grasping and arm lifting tasks with the most affected hand in patients with stroke and PD and the dominant hand for healthy participants. 3 conditions: MI, ME, and rest 3 blocks with 3 times each condition	EMG of deltoideus pars clavicularis, biceps brachii, extensor digitorum, flexor carpi radialis
Electrode recording		Fischer et al. (54)	Descriptive study	Experimental group: 10 (3♀), 61.3 (7) y, UPDRS-III off 43.5 (21.9)/on 17.9 (11.7), H&Y NI, surgical treatment	NI	NI	First part: gripping task at 15, 50, or 85% of the maximum sustainable force 3 blocks in each condition, with each block containing 3–5 trials for each hand and force level Second part: MI task of gripping 3 blocks with 3 trials per hand and a force level for each block	Monopolar Local Field Potentials (LFP) Gamma-beta power changes
PET scan	Joystick movement	Samuel et al. (57)	Descriptive study	Experimental group: 6 (NI), 62 (6) y, UPDRS off 24 (13), H&Y NI, pharmacological treatment Control group: 6 HS (3♀), 55 (4) y	NI	NI	Task: joystick movement 3 conditions: rest, MI, ME In conditions 1 and 2, relax hand loosely around the joystick Tested in an “off” state (12 h without medication)	Task performance (recall the last 4 movements) during MI and ME PET activation with rCBF
		Thobois et al. (55)	Descriptive study	Experimental group: 8 right-handed patients (3♀), 49.4 (5.3) y, UPDRS-III “off” 18.7 (6), H&Y 2 (0.5), dopaminergic treatment (6) or drug naive (2) Control group: 8 right-handed (5♀), 54 (12.8) y	Idiopathic PD (according to UK Brain Bank criteria) Positive and sustained response to dopaminergic treatment Asymmetric parkinsonian syndrome, affecting predominantly the right hemibody Prominent akinetic-rigid signs without tremor	NI	Task: sequential movement with a joystick 3 conditions: MI, ME, and rest 90 s/condition	Execution time PET activation with rCBF
PET scan	Joystick movement	Thobois et al. (56)	Descriptive study	Experimental group: 7 (1♀), 56.3 (11.4) y, UPDRS “on” 15.2 (8.5)/"off” 46.2 (15), H&Y NI, chronic electrical stimulation of the STN	NI	NI	Task: moving a joystick with the right hand in 3 sequential directions 6 conditions for a PET scan: rest without simulation, rest with effective unilateral left stimulation, ME without stimulation, ME with effective unilateral left stimulation, MI without stimulation, MI with unilateral left stimulation 2 times each condition Tested in an “off” state (12 h without medication)	Execution time STN rCBF changes during MI and ME
Clinical assessment	Various tasks of the upper limb	Yágüez et al. (39)	Clinical trial	Patients with Parkinson’s disease group: 12 (6♀), 67.0 (10.3) y, UPDRS NI, H&Y (range 1–3), pharmacological treatment Patients with Huntington’s disease group: 11 (5♀), 47.6 (10.0) y	NI	NI	Imagery training: imagine printed ideograms, imagine drawing them Physical practice: 4 sheets of drawing the ideograms	3 measurements (drawing ideograms): baseline, after imagery, and after physical practice Kinematic parameters: movement duration, tangential velocity Accuracy: heights, widths
		Sabaté et al. (40)	Descriptive study	Young-healthy group: 9 HS (NI), range: 20–38 y Mature-healthy group: 9 HS (NI), range: 40–65 y Patients with stroke group (3 years): 10 (NI), range: 44–66 y Patients with stroke group (32 weeks): 15 (NI), range: 41–72 y Patients with PD group: 8 (NI), range 54–64 y Patients with cerebellar stroke group: 8 (NI), range: 52–68 y Patients with osseous impairments group: 9 (NI), range: 17–42 y They were all right-handed	Being in good health	Obesity (>20% of ideal weight) Smokers	Task: sequence of 8 finger movements in a specific order Conditions: MI and ME 8 different sequences repeated 10 times for each hand	Execution time to perform each motor sequence 10 times Virtual delay
		Sabaté et al. (58)	Descriptive study	Patients with PD group: 10 (NI), range: 54–64 y, UPDRS NI, H&Y 1.8 (2.2), levodopa treatment Young healthy group: 15 (NI), range 24–49 y Mature-healthy group: 10 (NI), range: 50–72 y	NI	NI	3 tasks: (1) Slow cyclic movement: flexion-extension of the index finger at 40 movements per minute (2) Fast cyclic movement: same as (1) but as fast as possible (3) Continuous movement: turning a crank Conditions: tasks were realized in ME and MI, and auditory cues were added at times Tested in an “on” and “off” state	Task frequency Execution time
Clinical assessment Behavioral assessment	Various tasks of the upper limb	Bek et al. (59)	Descriptive study	Experimental group: 24 (9♀), 63.5 (6.34) y, UPDRS-III 38.4 (11.33), H&Y (range: 1–3), dopaminergic treatment for all except one Control group: 24 (13♀), 68.33 (5.38) y	NI	NI	AO: observation of a video, and patients were asked to imitate the action (moving their finger from one place to another) AO + MI: while watching, patients had to imagine what they would feel if they were the ones doing the movements 4 blocks of 30 trials each The first two blocks were AO, and the second two blocks were AO + MI Tested in an “on” state	Task-specific rating of visual and kinesthesic imagery with a short version of KVIQ (one after AO and one after AO + MI) Mean vertical amplitude
Imaging assessment (EMG, EEG, TMS)		Gündüz and Kiziltan (60)	Descriptive study	PD with apraxia group: 8 (3♀), 62.7 (13.4) y, UPDRS-III 13.8 (7.3), H&Y 1.9 (0.3), NI PD non-apraxia group: 11 (1♀), 55.2 (9.6) y, UPDRS-III 9.5 (3.5), H&Y 1.6 (0.5), NI Control group: 8 HS (2♀), 55.2 (8.6) y	NI	Disorders that could change the results of electrophysiological investigations contraindications to electrophysiological investigations suspicion of dementia	Task: thumb abduction with both arms 4 conditions: rest, MI, observation of an actor, ME 20 recordings Tested “under optimal dopaminergic treatment”	F-wave: amplitudes, onset latencies, persistence MEP responses: peak-to-peak amplitudes, onset latencies
		Tremblay et al. (61)	Descriptive study	Experimental group: 11 right-handed patients (5♀), 68.6 (5.8) y, UPDRS-III 23.4 (5.1), H&Y 2.4 (0.5), treatment NI Control group: 11 HS right-handed (8♀), 66.2 (4.9) y	NI	NI	4 video sequences of 10s each: REST task: relax with eyes closed OBS task: observe a sequence of scissoring action IMAG task: close eyes, mentally simulate scissoring action IMIT task: imitate the action 10 times per video Tested in an “on” state	MEP of FDI and ADM muscles in scissoring action Variation in MEP amplitude Variation in MEP latency VAS (0–10 cm): ease in imagining the action
		Cunnington et al. (62)	Descriptive study	Experimental group: 14 (0♀), 67.6 (10.5) y, UPDRS NI, H&Y 2.1 (0.9), pharmacological treatment Control group: 10 HS (0♀), 64.0 (8.9) y	NI	NI	Sequential button-pressing task 3 conditions: ME, MI, watching cues	Movement-related potentials: early component onset time, early slope, peak amplitude, peak time
Electrode recording		Kühn et al. (63)	Descriptive study	Experimental group: 8 (3♀), 57 (3) y, UPDRS on 12 (6.1)/off 38.1 (8.6), H&Y NI, dopaminergic treatment, STN surgery Subgroup of the experimental group: 5 patients	NI	NI	Experimental task: MI and ME of a warning-go reaction time task, subjects had to do a wrist extension Control task for the subgroup: imagine the face of a relative Tested in an “off” state	Subthalamic nucleus local field potential activity in beta frequency
fMRI	Verbal task	Péran et al. (64)	Descriptive study	Experimental group: 10 (NI), 60.3 (7.8) y, UPDRS off 30.1 (18.1)/on 15.7 (9.4), H&Y NI, dopamine agonists (levodopa)	Diagnosis of PD by a staff neurologist (according to UK Parkinson’s disease Brain Bank criteria) No history of other neurological or psychiatric disease	MMSE <25	3 tasks with a set of objects: object naming (ObjN), generation of an action word that could be realized with the object (GenA), mental simulation of this action (MSoA) Tested in an “on” and “off” state	Number of correct responses for ObjN + GenA BOLD for fMRI analysis
Behavioral assessment	Laterality judgment	Amick et al. (72)	Descriptive study	Experiment 1A: LPD: 15 (8♀), 66.0 (11.0) y, UPDRS NI, H&Y (range: 1.5–3), pharmacological treatment RPD: 12 (5♀), 59.9 (6.9) y, UPDRS NI, H&Y (range: 1.5–3), pharmacological treatment Control group: 13 HS (5♀), 62.7 (9.9) y Experiment 1B: a subset of 1A participants LPD: 7 (4♀), 61.7 (9.3) y, UPDRS NI, H&Y (Mdn = 2), NI RPD: 6 (4♀), 60.8 (10.5) y, UPDRS NI, H&Y (Mdn = 2.5), NI Control group: 6 HS (4♀), 62.3 (6.5) y	NI	NI	Experiment 1A: judging whether a pair of hands or objects are of the same laterality or not Experiment 1B: identical methods, except they performed only hand tasks and the hand to be mentally rotated was in the left visual field Tested in an “on” state	Primary outcome: number of errors Secondary outcome: response time
		Conson et al. (73)	Descriptive study	Experimental group: LPD group: 14 (6♀), 62.9 (4.7) y, UPDRS-III 12.9 (4.1), H&Y 1.9 (0.6), pharmacological treatment RPD group: 15 (4♀), 66 (8.6) y, UPDRS-III 15.4 (5.6), H&Y 1.7 (0.6), pharmacological treatment Control group: 30 HS (10♀), 49.7 (7.3) y	Diagnosis of idiopathic PD according to the United Kingdom Parkinson’s Disease Society brain bank Clinical and historical evidence of asymmetric motor disturbances Lack of PD-associated dementia (PDD) as diagnosed according to an algorithm for clinical diagnosis of PDD Lack of major depression	For patients with PD: PD patients with a total age-and educational-adjusted MMSE score (Italian version) <23.8 For HS: Diagnosis of PD or any other neurologic or psychiatric disorder Clinically evident dementia or major depression MMSE score below the normal cut-off	Laterality judgment experiment, 3 tasks: Patients had to tell whether the left or right hand of a human figure was marked, the human figure being front (task 1) or back (task 2) Patients performed a letter laterality judgment task (task 3) Each task included 48 trials Tested in an “on” state	Reaction times Accuracy
		Dominey et al. (50)	Descriptive study	Experimental group: 7 (3♀), 56.3 (8.0) y, UPDRS NI, H&Y (range: 1.5–2.5), pharmacological treatment, right side most affected Control group: 7 HS (2♀), 54.4 (11.7) y	Parkinson’s disease with predominant akinesia and no tremor Mainly, unilateral motor signs	NI	3 tasks in this experiment: (1) Touch the pad of each finger with the pad of the thumb alternately Three conditions for the task: motor task with visual control, motor task without visual control, and MI 12 combinations possible (left hand or right hand x 3 conditions x repeated 3 or 5 times) performed 5 times == > 60 trials (2.A) Judge if the letter presented was a mirror or normally oriented letter 32 trials in total (2 conditions x eight angles x two letters) (2.B) Determine if the hand presented is right or left hand (3) Imagine the upper-case letter corresponding to the lower-case letter presented and judge whether it is made of a straight line or has a curved line. 8 letters “straight” and 8 letters “curved” presented twice for 32 trials in total	(1) Execution time for each sequence (2.A/B) Reaction times (3) Percentage of the correct response and reaction time
Behavioral assessment	Laterality judgment	Scarpina et al. (74)	Descriptive study	Experimental group: RPD group: 10 (7♀), 65 (7) y, UPDRS-III 29.3 (11.1), H&Y NI, treatment NI LPD group: 10 (5♀), 61 (8) y, UPDRS-III 33 (14.93), H&Y NI, treatment NI Control group: 20 HS (9♀), 59 (8) y	NI	Other neurological conditions The presence of psychiatric syndromes or drug and alcohol abuse	2 tasks and their control tasks: Hand laterality task (HLT) Control: mirror letter discrimination tasks Mental motor chronometry (MMC) task in MI and ME: index and thumb opposition, thumb extension, middle finger crossed in the index, extension of the index and little finger Control: mental bar movement task Tested in “on” phase	Reaction time (RT) Accuracy Correlation between execution time for MI and execution time for ME
		Frak et al. (78)	Descriptive study	Experimental group: 8 (4♀), 59 (4.49) y, UPDRS NI, H&Y stage 3, L-dopa treatment Control group: 8 HS (3♀), 58 (5.08) y	NI	NI	Cylinder task: take a cylinder (with thumb and index) and pour water into another cylinder, then imagine and judge the feasibility of the grip presented Minimum 20 repetitions and 8 orientations for feasibility 50 times each Letter rotation task: judge whether a letter is in canonical or mirror form 42 stimuli, 2 times each	Cylinder task: preferred orientation of the opposition axis, feasibility level, and response time Letter rotation task: response time and accuracy
TMS		Van Nueunen et al. (65)	Descriptive study	Experimental group: 11 (5♀), 52.0 (7.8) y, UPDRS left side 1.1 (1.3)/UPDRS right side 7.6 (3.1), H&Y 1.4 (0.5), NI Control group: 12 HS (6♀), 61.3 (6.4) y	Idiopathic PD (according to UK Brain Bank criteria) Right-lateralized symptoms	MMSE <24 Other neurological disease Exclusion criteria for transcranial magnetic stimulation (epilepsy, pacemaker, implanted metal parts, cardiac arrhythmias)	Hand drawing laterality judgment task 4 postures for patients: both hands with palm up; left hand palms up; right hand palm down; left hand palm down; right hand palm up; both hands palm down. Posture is “matching” when the sides of the hand and laterality correspond Before each experimental session, subjects followed either a cTBS protocol over the right EBA or over the left PMd 2 sessions of 32 blocks with 12 trials/block each Tested in an “off” state	3 measurement sessions: baseline, after cTBS PMd, after cTBS EBA Reaction time Error rates Corticospinal excitability: MEP
fMRI		Helmich et al. (66)	Descriptive study	Main experiment: PD patient group: 19 (16♀), 53.2 (9.1) y, UPDRS-right 13.5 (5.0)/left 4.6 (2.8), H&Y 2.1 (0.5), treatment NI Control experiment: PD patients group (a part of the above-mentioned patients): 12 (4♀), 56.2 (10.0) y, UPDRS NI, H&Y NI, treatment NI Control group of right-handed: Elderly: 10 HS (4♀), 57.0 (6.2) y Young: 15 HS (8♀), 26.7 (3.3) y	For patients with PD: Idiopathic Parkinson’s disease (according to the UK Brain Bank criteria) Right-lateralized symptoms	Moderate–severe tremor MMSE <24 Other neurological diseases General exclusion criteria for MRI scanning	Main experiment: laterality judgment task of line drawing of right and left hands Patients had to change their arm position at each block 30 blocks of 16 trials each Control experiment: laterality judgment task for realistic photos of right and left hands Patients had to adopt one of the 4 postures requested at the beginning of each block 44 blocks of 8 trials each Tested in an “off” state	Reaction time Error rate fMRI: cerebral activation – beta values
fMRI	Laterality judgment	Helmich et al. (67)	Descriptive study	Tremulous patients with PD group: 18 (8♀), 56.7 (10.0) y, UPDRS-III 27.2 (8.1), H&Y 2 (0.3) Non-tremor patients with PD group: 20 (4♀), 59.1 (9.4) y UPDRS-III 27.9 (9), H&Y 2.1 (0.2) 12 without treatment and the rest with dopamine Control group: 19 HS (7♀), 58.6 (7.9) y	For patients with PD: Idiopathic PD is diagnosed according to the UK Brain Bank criteria Either clear presence or absence of resting tremor - Tremulous PD -- > UPDRS resting tremor score ≥ 2 for at least one hand during and an obvious history of resting tremor. - Non-tremor PD -- > UPDRS resting tremor score = 0 for each hand and no history of resting tremor	Clinical signs of dementia Other neurological diseases General exclusion criteria for MRI scanning	Laterality judgment task: right or left feet and hands in 4 different rotations and 2 different views 2 sessions of 30 min Tested in an “off” state	Reaction times Error rates fMRI: cerebral activation – beta values
Clinical assessment Behavioral assessment	Test and questionnaire	Heremans et al. (29)	Descriptive study	Experimental group: 14 (5♀), 59.1 (9.6) y, UPDRS 22.1 (11.5), H&Y 2.0 (0.8), pharmacological treatment Control group: 14 HS (6♀), 61.1 (6.6) y	NI	MMSE <24 Severe tremor Neurological comorbidity Unpredictable motor fluctuations Eye movement abnormalities Severe orthopedic problems of the upper limb Treatment with deep brain stimulation	GDAT: 3 conditions (ME, MI, rest) with 3 modalities (visual cues, auditory cues, no cues) 3 times each condition for all modalities Adapted BBT: 4 conditions (ME, MI with visual cues, MI with auditory cues, MI without cues) 3 times each condition Tested in an “on” state	Electrooculography: eye movement time, number, and amplitude Mental chronometry (for BBT only) VAS: 7-point scale: 1 = very hard, 7 = very easy
		Heremans et al. (68)	Descriptive study	Experimental group: 14 (5♀), 59.1 (9.6) y, UPDRS 22.1 (11.5), H&Y 2.0 (0.8), pharmacological treatment Control group: 14 HS (6♀), 61.1 (6.6) y	NI	MMSE <24 Severe tremor Neurological comorbidity Unpredictable motor fluctuations Severe orthopedic problems of the upper limb Treatment with deep brain stimulation	MIQ-R: questionnaire KVIQ: questionnaire CMIA: component 1 – hand rotation, component 2 – finger-thumb opposition accuracy, component 3 – finger-thumb opposition speed Adapted BBT: patients first performed the test and then imagined it, test perform Tested in an “on” state	Scores of MIQ-R, KVIQ, and CMIA Duration of ME and MI for BBT
Clinical assessment	Test and questionnaire	Gäumann et al. (42)	Longitudinal study	Patients with stroke: 25 (9♀), 63.3 (13.5) y Patients with multiple sclerosis: 25 (16♀), 51.0 (11.9) y Patients with Parkinson’s disease: 5 (0♀), 70.4 (3.3) y, NI	Diagnosis of stroke, multiple sclerosis, or Parkinson’s disease Age > 18 years MoCA >19 Being able to sit stable on an armless chair Being able to read and understand German	Persistent pain	MI ability: Body Rotation Task (BRT), Mental Chronometry (MC), KVIQ-20 MI perspective selection: patients were asked if they preferred an internal or external view based on the pictures they were shown, which were KVIQ items 4 measurement sessions in 2 weeks	Primary outcome: spontaneous MI perspective (internal, external)
		Peterson et al. (75)	Descriptive study	Experimental group: 28 (11♀), 71 (8.9) y, MDS-UPDRS-III on 26.6 (9.8)/off 37.6 (9.9), H&Y on 2.2 (0.4)/off 2.4 (0.3), levodopa treatment Control group: 32 HS (16♀), 70.3 (10.6) y	Diagnosis given by a certified neurologist	Severe orthopedic problems of upper/lower limbs Deep brain stimulation Other neurological disorder	KVIQ-20 Tested in an “on” and “off” state	Score of KVIQ-20
fMRI	Neurofeedback	Subramanian et al. (70)	Controlled trial	10 PD patients (4♀), range: 39–75 y, UPDRS NI, H&Y stage I-III, dopaminergic medication Experimental group: 5 Control group: 5	No history of psychiatric or other neurological problems No family history of PD	NI	Experimental group: MI strategy that proved useful for activating SMA during the initial assessment Control group: MI they used during the initial assessment Session 1: 2-6 M, 7 W, no duration specified Session 2: 2 W, 7 W, no duration specified	3 at W0, after session 1 and after session 2 Behavioral analysis: UPDRS, finger-tapping test fMRI analysis EMG analysis
		Tinaz et al. (33)	Non-RCT	Heartbeat counting task group: 10 (5♀), 62.6 (10.8), MDS-UPDRS 53.9 (12.3)/Part III 33.3 (8.3), H&Y 2.1 (0.1), stable treatment Neurofeedback group: 8 (4♀), 66.0 (8.5) y, MDS-UPDRS 44.8 (5.4)/Part III 32.1 (6.6), H&Y 2.0 (0), levodopa	Diagnosis of idiopathic PD according to the United Kingdom Parkinson’s Disease Society Brain Bank Clinical Diagnosis Criteria H&Y scale: ≤ stage 2.5 Stable dopaminergic medication	Not fully independent Neurological or psychiatric disorder A medical condition that might affect the central nervous system History of alcohol or illicit drug abuse Head injury resulting in loss of consciousness MoCA <21 Contraindications for an MRI	Heartbeat group: no task Neurofeedback group task: mindfulness body scan exercise and practice MI strategies that generated positive feedback during the initial testing. 3 W, every day, 10–15 min The heartbeat group was tested in off-state The neurofeedback group was tested after their first dose of medication	For neurofeedback group: 2 at baseline and after training MDS-UPDRS Part III Insula-dorsomedial frontal cortex functional connectivity (fMRI activity) For the heartbeat group: fMRI activity during heartbeat counting
PET scan	MI of whole body	Mori et al. (71)	Descriptive study	Experimental group: 10 (7♀), 57.1 (6.2) y, UPDRS-III 10.2 (2.3), H&Y 1.8 (0.4), naive TTT Control group: 12 HS (7♀), 9 right-handed and 3 left-handed, 51.2 (9.2) y	NI	For patients with PD: History of any kind of dopamine therapy For HS: Regular intake of medicines History of psychiatric or neurological diseases Contraindications to MRI and PET scanning	Supine position: (1) Stare at a marker of a human silhouette (2) MI of standing upright Standing position: (3) Stare at a target	rCBF

**Table 4:** Main results of non-randomized controlled trials and descriptive studies.

Assessment	Type	Articles	Evaluation	Main outcomes (comparison between groups, comparison between conditions)	*p-*value
Clinical assessment	MI of walking	Cohen et al. (43)	Passability experiment: passability estimation (% of body width) Imagery experiment: execution time	Passability estimation: PD-FOG vs. control group, no data available Passability estimation: PD-nonFOG vs. control group, no data available Passability estimation: PD-nonFOG vs. PD-FOG, no data available Execution time of walking in MI and ME across different door widths: PD-FOG vs. PD-nonFOG vs. control group, no data available Execution time of walking in MI and ME from different distances: PD-FOG vs. PD-nonFOG vs. control group, no data available Execution time of walking in ME by narrow doorway: PD-FOG vs. control group, no data available Execution time of walking in ME by narrow doorway: PD-FOG vs. PD-nonFOG, no data available	*p* = 0.01 *p* = 0.03 N.S. N.S. N.S. *p* < 0.001 *p* < 0.001
		Ehgoetz Martens et al. (44)	Experiment 1: magnitude of error Experiment 2: execution time	Absolute error of pointing and walking judgment: PD-FOG group vs. PD-nonFOG group, no data available Execution time for ME of walking: PD-nonFOG group vs. PD-FOG group, no data available Execution time for MI task: PD-nonFOG group vs. PD-FOG group, no data available	*p* = 0.01 *p* = 0.03 N.S.
fMRI		Huang et al. (47)	BOLD response	BOLD response during MI of normal gait of bilateral SMA, right superior temporal, and right medial superior frontal gyrus: PD-nonFOG group vs. control group, no data available BOLD response during MI of FOG gait of bilateral frontal lobe, left superior temporal lobe, right insula: PD-FOG vs. PD-nonFOG, no data available	*p* = 0.04 *p* = 0.05
		Maidan et al. (48)	Neural brain activation	Activation in frontal, parietal, temporal, and occipital lobes during MI of walking on usual path compared to watching: experimental group vs. control group, no data available Activation in frontal and occipital lobes during MI of obstacle walking compared to watching: experimental group vs. control group, no data available Activation in left parietal and right frontal lobes during MI of walking while navigating compared to watching: experimental group vs. control group, no data available	*P* = 0.04 *p* = 0.09 *p* = 0.05
		Peterson et al. (28)	Execution time BOLD with a region of interest	Execution time, ME of tasks, experimental group vs. control group, no data available Execution time, MI of tasks, experimental group vs. control group, no data available Brain activity in left globus pallidus, experimental group vs. control group, no data available	*p* < 0.001 N.S. *p* < 0.001
		Snijders et al. (46)	Execution time (imagery task) Gait data (step length, gait asymmetry) ROI analysis for fMRI	Normalized step-length, experimental group 0.71 (0.08) vs. control group 0.78 (0.08) Normalized step-length, FOG 0.66 (0.15) vs. nonFOG 0.73 (0.07) Gait asymmetry, experimental group 0.036 (0.027) vs. control group 0.015 (0.011) Gait asymmetry, FOG 0.040 (0.027) vs. nonFOG 0.033 (0.029) Execution time on MI tasks, experimental group vs. control group, no data available Execution time on MI tasks, FOG vs. nonFOG, no data available fMRI activity in mesencephalic locomotor region, FOG vs. nonFOG, no data available	*p* = 0.01 *p* = 0.17 *p* < 0.001 *p* = 0.50 *p* = 0.35 *p* = 0.07 *p* = 0.05
PET scan		Maillet et al. (51)	Behavioral session: KVIQ score, execution time PET session: execution time, regional cerebral blood flow (rCBF)	Behavioral session: Execution time of walking in MI and ME in all conditions: experimental group off vs. control group, no data available Execution time of walking in MI and ME in all conditions (except 6 m*9 cm): experimental group on vs. control group, no data available PET session: rCBF during MI of walking compared to control task in left caudal SMA, lateral PMC, right dACC, SPL, pontomesencephalic area: experimental group off vs. control group, no data available rCBF during MI of walking compared to control task in pre-SMA, DLPFC, left dACC, right M1, S1, lateral PMC, insula, thalamus, putamen, cerebellum, red nucleus: experimental group off vs. control group, no data available	*p* = 0.03 N.S. *p* < 0.001 *p* < 0.001
		Weiss et al. (49)	MI of walking distance Walking distance Stride length Velocity PET activation with rCBF	Walking distance: STN-DBS ON 94.7 (15.4) m vs. STN-DBS OFF 62.6 (27.2) m Gait velocity: STN-DBS ON 1.1 (0.2) m/s vs. STN-DBS OFF 0.7 (0.3) m/s Mean stride length: STN-DBS ON 56.2 (8.8) cm vs. STN-DBS OFF 43.2 (14.9) cm Correlation between MI of walking distance and MI execution time while STN-DBS OFF: 30s 24.6 (11.8) m vs. 60s 36.6 (23.2) m vs. 90s 49.2 (27.0) m Correlation between MI of walking distance and MI execution time while STN-DBS ON: 30s 42.0 (25.8) m vs. 60s 62.3 (23.5) m vs. 90s 84.8 (37.0) m MI of walking distance 30s, 60s, and 90s: STN-DBS ON vs. STN-DBS OFF, no data available Neural activity increase in SMA, right SPL: imagery of gait vs. imagine stance, no data available	*p* < 0.001 *p* < 0.001 *p* < 0.001 *p* < 0.05 *p* < 0.01 *p* < 0.05 *p* < 0.05
Behavioral assessment	Thumb opposition	Avanzino et al. (51)	Temporal error Interval reproduction accuracy index	Temporal error during SYNC 0.5 Hz: experimental group vs. control group, no data available Temporal error during CONT-EXE 0.5 Hz: experimental group vs. control group, *no data available* Temporal error during CONT-MI 0.5 Hz: experimental group vs. control group, *no data available* Temporal error during all conditions 1.5 Hz: experimental group vs. control group, no data available Interval reproduction accuracy index during SYNC 0.5 Hz: experimental group vs. control group, no data available Interval reproduction accuracy index during CONT-EXE 0.5 Hz: experimental group vs. control group, no data available Interval reproduction accuracy index during CONT-MI 0.5 Hz: experimental group vs. control group, no data available Interval reproduction accuracy index during all conditions 1.5 Hz: experimental group vs. control group, no data available	*p* = 0.79 p = 0.05 *p* = 0.04 N.S. *p* = 0.47 *p* = 0.05 *p* = 0.03 N.S.
PET scan		Cunnington et al. (52)	Relative rCBF	rrCBF, Parkinson’s disease “off” state: medial frontal gyrus (SMA): imagine 62.9 vs. rest 60.6 rrCBF, Parkinson’s disease “off” state: right lateral premotor: imagine 60.4 vs. rest 58.7 rrCBF, Parkinson’s disease “off” state: right inferior parietal lobule: imagine 55.2 vs. rest 53.1 rrCBF, Parkinson’s disease “on” state: medial frontal gyrus (SMA): imagine 61.9 vs. rest 59.7 rrCBF, Parkinson’s disease “on” state: right inferior parietal lobule: imagine 49.6 vs. rest 47.5	*p* < 0.001 *p* < 0.001 *p* < 0.001 *p* < 0.001 *p* < 0.001
Electrode recording		Leiguarda et al. (53)	Firing rate of the globus pallidus internus	Firing rate: rest 77.82 Hz vs. MI 39.37 Hz Firing rate: rest 77.82 Hz vs. movement execution 55.50 Hz	*p* = 0.04 *p* = 0.07
EMG	Hand gripping	Kobelt et al. (41)	EMG of deltoideus pars clavicularis, biceps brachii, extensor digitorum, flexor carpi radialis	EMG showed activation during MI in 2 of 5 patients with PD EMG, deltoideus pars clavicularis activation: MI vs. rest, no data available EMG, biceps brachii activation: MI vs. rest, no data available EMG, extensor digitorum activation: MI vs. rest, no data available EMG, flexor carpi radialis activation: MI vs. rest, no data available	NA *p* < 0.001 *p* = 0.01 N.S. N.S.
Electrode recording		Fischer et al. (54)	Monopolar Local Field Potentials (LFP) Gamma-beta power changes	Beta change in early window during imagined grips, low force level vs. rest, no data available Beta change in early window during executed grips, low force level vs. rest, no data available Beta change in early window during imagined grips, medium force level vs. rest, no data available Beta change in early window during executed grips, medium force level vs. rest, no data available Beta change in early window during imagined grips, high force level vs. rest, no data available Beta change in early window during executed grips, high force level vs. rest, no data available Gamma change in early window during imagined grips, low force level vs. rest, no data available Gamma change in early window during executed grips, low force level vs. rest, no data available Gamma change in early window during imagined grips, medium force level vs. rest, no data available Gamma change in early window during executed grips, medium force level vs. rest, no data available Gamma change in early window during imagined grips, high force level vs. rest, no data available Gamma change in early window during executed grips, high force level vs. rest, no data available	N.S. *p* < 0.001 *p* = 0.01 *p* < 0.001 *p* = 0.05 *p* < 0.001 N.S. *p* = 0.05 *p* = 0.05 *p* = 0.01 *p* = 0.01 *p* = 0.05
PET scan	Joystick movement	Samuel et al. (57)	Task performance (recall the last 4 movements) in MI/ME PET activation with rCBF	Median number of recalled imagery movements, experimental group 3.7 (range: 3–4) vs. control group 3.7 (range: 3–4) Median number of recalled executed movements, experimental group 3.2 (range: 0–4) vs. control group 3.3 (range: 0–4) Response time, experimental group 0.85 (0.3) s vs. control group 0.46 (0.1) s Activity during the MI task in dorsolateral and mesial frontal cortex, experimental group vs. control group, no data available Activity during the ME task in right dorsolateral frontal cortex and basal ganglia, experimental group vs. control group, no data available	*p* = 0.50 *p* = 0.43 *p* = 0.01 *p* < 0.01 *p* < 0.01
		Thobois et al. (55)	Execution time PET activation with rCBF	Execution time, MI of the experimental group, left hand: 5245 (1840) ms vs. right hand: 5882 (1863) ms Execution time, ME of the experimental group, left hand: 5109 (1278) ms vs. right hand: 5925 (1734) ms Experimental group, rCBF increase in bilateral superior parietal lobe/left anterior cingulate cortex/left lateral premotor cortex/left inferior frontal gyrus/left DLPFC/occipital cortex, MI of left hand vs. rest, no data available Experimental group, rCBF increase in left lateral premotor cortex/SMA/bilateral superior parietal lobe/DLPFC/right primary motor cortex, MI of right hand vs. rest, no data available Control group, rCBF increase in bilateral superior parietal lobe/supplementary motor area/left lateral premotor cortex/inferior frontal gyrus/dorsolateral prefrontal cortex/right cerebellar hemisphere, MI of left hand vs. rest, no data available Control group, rCBF increase in left primary motor cortex/lateral premotor cortex/SMA, DLPFC/superior parietal lobe/right cerebellar hemisphere, MI of right hand vs. rest, no data available	*p* < 0.05 *p* < 0.05 *p* < 0.05 *p* < 0.05 *p* < 0.05 *p* < 0.05
PET scan	Joystick movement	Thobois et al. (56)	Execution time STN rCBF changes during MI and ME	Execution time, ME vs. MI, no data available Execution time, STN on 4.74 s vs. STN off 5.76 s rCBF activation in left primary motor cortex and SMA without stimulation, ME vs. rest, no data available rCBF activation in dorsolateral prefrontal cortex and SMA without stimulation, MI vs. rest, no data available rCBF activity increased in the bilateral prefrontal cortex, left thalamus, and putamen with stimulation, ME with stimulation vs. ME without stimulation, no data available rCBF activity decreased in right primary motor cortex, inferior parietal lobe and SMA with stimulation, ME with stimulation vs. ME without stimulation, no data available rCBF activity increased in bilateral dorsolateral prefrontal cortex, left thalamus and putamen, MI with stimulation vs. MI without stimulation, no data available rCBF activity decreased in left SMA and primary motor cortex, MI with stimulation vs. MI without stimulation, no data available	*p* = 0.23 *p* = 0.07 *p* < 0.05 *p* < 0.05 *p* < 0.05 *p* < 0.05 *p* < 0.05 *p* < 0.05
Clinical assessment	Various tasks of the upper limb	Yágüez et al. (39)	3 measurements (drawing ideograms): baseline, after imagery, and after physical practice Kinematic parameters: execution time, tangential velocity Accuracy: heights, widths	Small ideograms, PD patient movement duration: baseline vs. post-imagery, no data available Small ideograms, PD patient movement duration: post-imagery vs. post-practice, no data available Small ideograms, PD patient movement duration: baseline vs. post-practice, no data available Large ideograms, PD patient movement duration: baseline vs. post-imagery, no data available Large ideograms, PD patient movement duration: post-imagery vs. post-practice, no data available Large ideograms, PD patient movement duration: baseline vs. post-practice, no data available Height and width of small and large ideograms for PD patients: baseline vs. post-imagery, no data available	N.S. *p* = 0.03 *p* = 0.01 N.S. N.S. N.S. N.S.
		Sabaté et al. (40)	Execution time to perform each sequence 10 times Virtual delay	Execution time for ME: patients with PD group vs. mature-healthy group, no data available Execution time for MI: patients with PD group vs. mature-healthy group, no data available Virtual delay: patients with PD group vs. mature-healthy group, no data available	*p* < 0.001 *p* < 0.001 N.S.
		Sabaté et al. (58)	Task frequency Execution time	Execution time for a slow cyclic task, ME vs. MI, no data available Execution time for a fast cyclic task, ME vs. MI, no data available Execution time for the slow continuous movement task, ME vs. MI, no data available	*p* = 0.39 *p* < 0.001 *p* < 0.001
Clinical assessment Behavioral assessment		Bek et al. (59)	Task-specific rating of visual and kinesthesic imagery with short version of KVIQ (one after AO and one after AO + MI) Mean vertical amplitude	Mean vertical amplitude after AO: experimental group vs. control group, no data available Mean vertical amplitude after AO + MI: experimental group vs. control group, no data available Task-specific rating of visual and kinesthesic imagery before MI instructions: experimental group vs. control group, no data available Task-specific rating of visual and kinesthesic imagery after MI instructions: experimental group vs. control group, no data available	*p* = 0.09 *p* = 0.07 N.S. N.S.
Imaging assessment (EMG, EEG, TMS)		Gündüz and Kiziltan (60)	F-wave: amplitudes, onset latencies, persistence MEP responses: peak-to-peak amplitudes, onset latencies	Mean amplitude F-waves in control group, imagination vs. rest, no data available Mean amplitude F-waves in PD non-apraxia group, imagination vs. rest, no data available Mean amplitude F-waves in PD with apraxia group, imagination vs. rest, no data available	*p* = 0.03 *p* = 0.01 N.S.
		Tremblay et al. (61)	MEP of FDI and ADM muscles in scissoring action Variation in MEP amplitude Variation in MEP latency VAS (0–10 cm): ease in imagining the action	VAS: experimental group 6.5 (0.7) cm vs. control group 7.1 (0.6) cm FDI MEP amplitude in experimental group: REST vs. IMAG, no data available FDI MEP amplitude in control group: REST vs. IMAG, no data available ADM MEP amplitude in experimental group: REST vs. IMAG, no data available ADM MEP amplitude in control group: REST vs. IMAG, no data available FDI MEP latency in experimental group: REST vs. IMAG, no data available FDI MEP latency in control group: REST vs. IMAG, no data available ADM MEP latency in experimental group: REST vs. IMAG, no data available ADM MEP latency in control group: REST vs. IMAG, no data available	*p* = 0.50 N.S. *p* < 0.01 N.S. *p* < 0.05 N.S. N.S. N.S. *p* < 0.01
Imaging assessment (EMG, EEG, TMS)	Various tasks of the upper limb	Cunnington et al. (62)	Movement related potentials (MRP): early component onset-time, early slope, peak amplitude, peak time	MRP onset times at position Cz: experimental group 1.64 (0.54) s vs. control group 1.70 (0.49) s MRP early slope: experimental group vs. control group, no data available MRP peak amplitude: experimental group vs. control group, no data available MRP peak times: experimental group 75 (195) ms vs. control group 109 (187) ms	N.S. *p* < 0.001 *p* < 0.05 N.S.
Electrode recording		Kühn et al. (63)	Subthalamic nucleus local field potential activity in beta frequency	Mean beta ERD change from baseline following auditory cue: ME 44.6% (6.4) vs. MI 36.7% (4.5) Mean beta ERD change from baseline: ME vs. control task, no data available Mean beta ERD change from baseline: MI vs. control task, no data available	*p* = 0.13 *p* < 0.01 *p* < 0.001
fMRI	Verbal task	Péran et al. (64)	Number of correct responses for ObjN + GenA BOLD for fMRI analysis	Brain activation in prefrontal cortex bilaterally and in the parietal–occipital junction bilaterally, ObjN vs. MSoA, no data available	*p* < 0.001
Behavioral assessment	Laterality judgment	Amick et al. (72)	Primary outcome: number of errors Secondary outcome: response time	Experiment 1A: Hand errors: RPD vs. control group, no data available Hand errors: LPD vs. control group, no data available Object errors and RT: RPD vs. LPD vs. control group, no data available Experiment 1B: Hand errors: LPD vs. control group, no data available Hand errors: LPD vs. RPD, no data available	*p* = 0.01 *p* = 0.90 N.S. *p* = 0.01 *p* = 0.02
		Conson et al. (73)	Reaction times Accuracy	Accuracy: LPD group vs. RPD group vs. control group, no data available Reaction times: LPD group vs. RPD group vs. control group, no data available Reaction times for all groups: left-marked front-facing bodies vs. right-marked front-facing bodies, no data available Reaction times for LPD group: left-marked back-facing bodies vs. right-marked back-facing bodies, no data available Reaction times for RPD group: right-marked back-facing bodies vs. left-marked back-facing bodies, no data available Reaction times for control group: left-marked back-facing bodies vs. right-marked back-facing bodies, no data available	*p* = 0.49 *p* = 0.95 N.S. *p* = 0.01 *p* = 0.03 N.S.
		Dominey et al. (50)	(1) Execution time for each sequence (2.A/B) Reaction time (RT) (3) Percentage of the correct response and reaction time	Experiment 1: Execution time: experimental group 29.73 s vs. control group 17.51 s Execution time: both groups right hand 25.16 s vs. both groups left hand 22.10 s Execution time: experimental group right hand 32.87 s vs. experimental group left hand 26.60 s Execution time: control group right hand 17.44 vs. control group left hand 17.59 Experiment 2: RT: experimental group 1925 ms vs. control group 1,614 msec Experiment 3: Percentage of correct response: experimental group vs. control group, no data available RT: experimental group vs. control group, no data available	*p* < 0.001 *p* = 0.05 NI NI *p* < 0.001 N.S. N.S.
		Scarpina et al. (74)	Reaction time (RT) Accuracy Correlation between execution time for MI and execution time for ME	RT (z-score), hand laterality task, right group 0.17 (0.66) vs. left group 0.14 (0.78) vs. control group 0.08 (0.76) Accuracy, hand laterality task, right group 68.75 (23.01) vs. left group 74.58 (25.72) vs. control group 76.14 (19.11) RT (z-score), mental letter discrimination task, right group-0.038 (0.78) vs. left group-0.013 (0.81) vs. control group-0.022 (0.89) Accuracy, mental letter discrimination task, right group 68.75 (23.01) vs. left group 74.58 (25.72) vs. control group 76.14 (19.11) Accuracy, mental letter discrimination task, right group 68.75 (23.01) vs. control group 76.14 (19.11) Execution time (z-score), mental bars movement task, right group vs. control group, no data available Execution time (z-score), mental bars movement task, left group vs. control group, no data available Execution time (z-score), mental bars movement task, right group vs. left group, no data available	*p* = 0.78 *p* = 0.53 *p* = 0.96 *p* = 0.02 *p* = 0.03 *p* = 0.02 *p* = 0.58 *p* = 0.13
		Frak et al. (78)	Cylinder task: preferred orientation of the opposition axis, feasibility level, and response time (RT) Letter rotation task: response time (RT) and accuracy	Cylinder task, RT: experimental group 1779 (425) ms vs. control group 1648 (458) ms Letter rotation task, RT: experimental group vs. control group, no data available Letter rotation task, number of errors: experimental group vs. control group, no data available	*p* > 0.50 *p* > 0.80 *p* > 0.60
TMS		Van Nueunen et al. (65)	3 measurement sessions: baseline, after cTBS PMd, after cTBS EBA Reaction time (RT) Error rates Corticospinal excitability: MEP	Baseline error rates: experimental group 3.3% (1.1) vs. control group 2.3% (0.7) Baseline reaction times: experimental group 1194 (97) ms vs. control group 1257 (81) ms Difference in RT between matching and non-matching posture in the experimental group, baseline vs. EBA-cTBS, no data available Difference in RT between matching and non-matching posture in the control group, baseline vs. PMD-cTBS, no data available	*p* = 0.69 *p* = 0.62 *p* = 0.03 N.S.
fMRI		Helmich et al. (66)	Reaction time Error rate fMRI: cerebral activation – beta values	Cerebral activity of EBA and OPC, rotation-related effects, right-hand vs. left-hand, no data available Main experiment Reaction times: left hand 1549 (102) ms vs. right hand 1527 (97) ms Error rates: left hand 7% (1) vs. right hand 8% (1) Control experiment Reaction times: PD patient group 1547 (126) ms vs. elderly 1178 (123) ms vs. young 1006 (76) ms Error rates: PD patient group 11% (2) vs. elderly 4% (2) vs. young 4% (1)	*p* < 0.05 N.S. N.S. *p* < 0.001 *p* = 0.01
		Helmich et al. (67)	Reaction times Error rates fMRI: cerebral activation – beta values	Cerebral activity in B3a, tremulous PD vs. control group, and non-tremor PD patients, no data available Reaction times: tremulous PD patients vs. non-tremor PD patients vs. control group, no data available Error rates: tremulous PD patients 11.7% (7.9) vs. non-tremor PD patients 14.0% (9.6) vs. control group 7.7% (6.1)	*p* < 0.01 *p* = 0.87 N.S.
Clinical assessment Behavioral assessment	Test and questionnaire	Heremans et al. (29)	Electrooculography: eye movement time, number, and amplitude Mental chronometry (for BBT only) VAS: 7-point scale: 1 = very hard, 7 = very easy	Eye movement time during GDAT: experimental group 369 (164) ms vs. control group 271 (141) ms Eye movement time during GDAT in rest condition: experimental group vs. control group, no data available Mental chronometry during BBT: experimental group 28.4 (6.5) s vs. control group 23.2 (4.9) s Mental chronometry during BBT for all subjects: ME vs. MI with visual cues, no data available Mental chronometry during BBT for all subjects: ME vs. MI without cues, no data available VAS during GDAT for all subjects: no cues vs. visual cues and auditory cues, no data available VAS during BBT: for all subjects: no cues vs. visual cues and auditory cues, no data available	*p* < 0.05 N.S. *p* < 0.02 N.S. *p* < 0.05 *p* = 0.03 *p* = 0.03
		Heremans et al. (68)	Scores of MIQ-R, KVIQ-20, and CMIA Duration of ME and MI for BBT	MIQ-R, total score: experimental group 4.8 (1.7) vs. control group 5.6 (1.4) KVIQ-20, total score: experimental group 2.5 (1.1) vs. control group 2.0 (2.1) CMIA component 1, total accuracy: experimental group 83.9% (9.6) vs. control group 84.7% (9.0) CMIA component 2, total score: experimental group 2.8 (0.7) vs. control group 2.9 (0.3) CMIA component 3, execution time in ME: experimental group 58.4 (14.3) s vs. control group 66.1 (15.4) s CMIA component 3, execution time in MI: experimental group 55.9 (21.8) s vs. control group 63.1 (18.5) s BBT, execution time in ME: experimental group 25.7 (4.2) s vs. control group 19.7 (2.7) s BBT, execution time in MI: experimental group 32.2 (8.6) s vs. control group 27.6 (6.3) s	N.S. N.S. N.S. N.S. N.S. N.S. *p* < 0.01 *p* < 0.01
Clinical assessment		Gäumann et al. (42)	Primary outcome: spontaneous MI perspective (internal, external)	Mean perspective preference during KVIQ visual subscale: internal 71.5% vs. external 26.3% vs. both 0.4% vs. not possible 2.3% Mean perspective preference during KVIQ kinesthetic subscale: internal 73.3% vs. external 25.2% vs. both 0.3% vs. not possible 1.4%	NI NI
		Peterson et al. (75)	Score of KVIQ-20	KVIQ-20, experimental group “on” 68.1 (23.3) vs. experimental group “off” 65.8 (22.0) KVIQ-20, experimental group “off” 65.8 (22.0) vs. control group 72.2 (20.6) KVIQ-20, experimental group “on” 68.1 (23.3) vs. control group 72.2 (20.6)	p = 0.15 *p* = 0.25 p = 0.46
fMRI	Neurofeedback	Subramanian et al. (70)	3 at W0, after session 1 and after session 2 Behavioral analysis: UPDRS, finger-tapping test fMRI analysis EMG analysis	UPDRS, experimental group pre-14.2 vs. experimental group post-9 UPDRS, control group pre-15 vs. control group post-13.4 Finger tapping test on affected hand, experimental group pre-210.6 vs. experimental group post-266.2 Finger tapping test on affected hand, control group pre-177 vs. control group post-178.2 SMA fMRI activity in localizer block, experimental group vs. control group, no data available SMA fMRI activity in experimental group, neurofeedback vs. control testing, no data available SMA fMRI activity in control group, neurofeedback vs. control testing, no data available	p = 0.04 *p* = 0.34 p = 0.04 p = 0.69 *p* = 0.26 p = 0.50 p = 0.04
		Tinaz et al. (33)	For neurofeedback group: 2 at baseline and after training MDS-UPDRS part III Insula-dorsomedial frontal cortex functional connectivity (fMRI activity) For heartbeat group: fMRI activity during heartbeat counting	MDS-UPDRS-III, neurofeedback group pre-32.1 (6.6) vs. neurofeedback group post-31.8 (4.5) fMRI activity in right insula and dorsomedial frontal cortex in heartbeat group, no data available Insula dorsomedial frontal cortex connectivity (z-score), neurofeedback group pre-0.15 (0.36) vs. neurofeedback group after post-0.19 (0.27)	*p* = 0.87 *p* = 0.05 *p* = 0.01
PET scan	MI of whole body	Mori et al. (71)	rCBF	rCBF responses during standing position in right cerebellar vermis and left paracentral gyrus, experimental group vs. control group, no data available rCBF responses during standing position in bilateral middle frontal gyrus, experimental group vs. control group, no data available rCBF responses during MI of standing, experimental group vs. control group, no data available	*p* = 0.05 *p* = 0.05 N.S.

#### 3.3.1 Participants’ characteristics

In most of these studies (39–41), patients with PD were compared with HS of the same age. The mean (SD) number of participants per study was 30 (±18) and the mean age was 61 (±8). The groups were comprised of an average of 35.5% women and 64.5% men. For patients with PD, the main inclusion criteria were a diagnosis of idiopathic PD (10 studies specified that the diagnosis was made using the UK brain bank criteria) and an H&Y score. A total of 21 out of 41 studies did not mention the inclusion criteria. Four studies included patients with other neurological conditions, such as stroke, multiple sclerosis, and Huntington’s disease (39–42).

The Kinesthetic and Visual Imagery Questionnaire (KVIQ) was used to evaluate the ability of subjects to imagine from a first-person perspective by assessing the clarity of the image (visual: V subscale) and the intensity of the sensations (kinesthetic: K subscale) (28, 29, 42).

#### 3.3.2 Protocols

We have grouped the studies according to whether they concern the lower limb, the upper limb, or language-related MI exercises. Subgroups were created within each category.

Eight studies focused on the lower limb using the MI of walking. Among these studies, the protocols were heterogeneous. Five studies tested MI walking in a straight line with different distances ranging from 2 to 15 m; 2 studies tested MI walking in a straight line, turning, turning back; and 1 study tested walking on an obstacle path.

The upper limb was involved in 16 studies. Three studies tested a thumb opposition task, 2 studies tested hand gripping, 3 studies tested joystick movement, and 8 studies tested various upper limb tasks with 8 different interventions.

Language-related tasks (verbal tasks) were used in only one study. Finally, other studies did not fit into the three categories mentioned above. Eight studies performed lateral judgment tasks, five used MI tests and questionnaires, two tested neurofeedback, and one tested whole-body MI.

Not all studies have evaluated patients with PD under the same conditions. Eleven studies evaluated patients during their off phase, 10 during the on phase, 6 during both phases, and 14 did not mention this information.

#### 3.3.3 Outcomes for lower limb

Of these studies, 2 assessed walking in clinical conditions (40, 41); execution time was also used (7 studies) during different tasks (28, 43–48); and 6 assessed brain activity with regional Cerebral Blood Flow (rCBF) using a Positron Emission Tomography (PET) scan (45, 49) as well as using functional Magnetic Resonance Imaging (fMRI) (25, 44, 45, 49).

#### 3.3.4 Outcomes for upper limb

In the thumb-opposition studies, Dominey et al. (50) evaluated the execution time for MI and ME. Avanzino et al. (51) assessed the timing error rate. Cunnington et al. (52) performed this task under a PET scan and compared the rCBF. Leiguarda et al. (53) analyzed the firing rate of the globus pallidus internus using microelectrode recording.

For hand gripping, muscle activation by electromyography (EMG) and monopolar local field potentials were evaluated (41, 54).

All joystick movement studies were conducted using a PET scan (55–57). In addition, two of them evaluated the execution time (55, 56).

For studies with varied upper limb tasks, the evaluations were also heterogeneous. The execution time was evaluated in three studies (39, 40, 58); KVIQ was assessed in one study (56); F-waves were assessed by EMG (59, 60); the amplitude of motor evoked potential by transcranial magnetic stimulation (TMS) (60, 61); movement-related potentials by electroencephalogram (62); and local field potentials by electrode recording (63).

#### 3.3.5 Outcomes for verbal tasks

Péran et al. (64) used the number of correct responses and an fMRI as a means of assessment.

#### 3.3.6 Outcomes for laterality judgment

Reaction time and error rate were measured for all these studies. The motor evoked potentials (MEP) amplitude was measured using TMS (65). An fMRI was used in two studies (66, 67).

#### 3.3.7 Outcomes for MI tests and questionnaire

Several tests were used in the various studies. The score of these studies was used as an outcome. The KVIQ, Motor Imagery Questionnaire-Revised (MIQ-R), the Gait Imagery Questionnaire (GIQ), and the Chaotic Motor Imagery Assessment were used. The execution time was also measured for the BBT (29, 68).

#### 3.3.8 Outcomes for neurofeedback intervention

In these non-RCT studies, the fMRI and UPDRS scores were used (69, 70).

#### 3.3.9 Outcomes for MI of the whole body

The rCBF was assessed by using a PET scan (71).

#### 3.3.10 Main results for lower limb (8 studies: 257 participants)

First, regarding imagined execution of walking time, three studies showed that there was no significant difference between PD and HS-MI (28, 44, 46). Cohen et al. (43) also found no significant difference between patients with PD with and without freezing of gait (FOG).

Second, regarding execution time of walking for PD/HS-ME, Peterson et al. (28) showed that patients with PD are slower than patients with HS (*p* < 0.001). It has been shown that patients with FOG were slower than patients without FOG in normal walking (*p* = 0.03) and when walking through a narrow doorway (*p* < 0.001) (43, 44).

Maillet et al. (45) investigated the influence of levodopa on the neural networks involved in the MI of gait in advanced PD and found that patients in the *off* phase had significantly different durations during the MI of gait compared to HS (*p* < 0.03), while in the *on* phase there was no significant difference when compared to HS. Weiss et al. (49) assessed the disparity between active and inactive transcranial stimulation in patients. When stimulation was active and for the MI condition, patients walked 51% further (*p* < 0.001), 57% faster (*p* < 0.001), and took 30% longer steps (*p* < 0.001).

Regarding brain activity, Maillet et al. (45) observed that MI of walking in patients with PD compared to HS increased brain activation in the premotor-parietal cortices and pontomesencephalic tegmentum and decreased brain activation in the motor and frontal associative areas, basal ganglia, thalamus, and cerebellum. Maidan et al. (48) found that compared to HS, patients with PD had higher activation in the frontal, parietal, temporal, and occipital lobes during MI of usual walking (*p* < 0.04). Huang et al. (47) demonstrated that during walking with MI, compared to controls, patients with PD without FOG had more brain activity in bilateral supplementary area, right superior temporal, and right medial superior frontal gyrus (*p* < 0.04). Weiss et al. (49) showed that, with or without deep brain stimulation in the subthalamic nucleus, the MI of walking induced activity in the supplementary motor area and the right superior parietal lobule against a rest condition (*p* < 0.05). In terms of the difference in FOG, Snijders et al. (46) found that FOG patients exhibited increased brain activity on fMRI in the mesencephalic locomotor region during MI of gait compared to non-FOG patients (*p* < 0.05).

#### 3.3.11 Main results for the thumb-opposition task (4 studies: 52 participants)

The Dominey et al. (50) study showed that patients with PD were 69.8% slower compared to HS in the execution time of the thumb-opposition task (MI and ME data combined) (*p* < 0.001). Avanzino et al. (51) found that when the task was performed in a 0.5 Hz timing and the auditory cue was removed, patients with PD made more errors when continuing the task in both MI (*p* = 0.04) and ME (*p* = 0.05) conditions, which was not the case for a 1.5 Hz timing. In the study by Cunnington et al. (52), it was observed that the level of activation in the supplementary motor area followed a typical pattern in patients with PD when they were both in the “*off*” and “*on*” medication states during MI compared to the resting state (*p* < 0.001).

#### 3.3.12 Main results for hand gripping task (2 studies: 32 participants)

Kobelt et al. (41) conducted a study on patients with stroke and PD by measuring their muscle activity by EMG. Their findings showed a significant activation of the deltoideus pars clavicularis (*p* < 0.001) and biceps brachii (*p* = 0.01) during the hand gripping task in MI in comparison to a resting state. There was, however, no significant difference in activation between MI and rest in the extensor digitorum and flexor carpi radialis muscles. Fischer et al. (54) recorded local field potentials with TMS in PD patients. They found that beta activity decreased significantly for MI at the two highest force levels compared to rest (range: *p* < 0.01–0.05) and for ME at all force levels (*p* < 0.001); gamma activity increased significantly at MI at the two highest force levels again compared to rest (range: *p* < 0.01–0.05) and for ME at all force levels (range: *p* < 0.01–0.05).

#### 3.3.13 Main results for joystick movement (3 studies: 35 participants)

Thobois et al. (55) observed that patients with PD performed the joystick movement task slower with their more affected side than with their other side in both the MI and ME conditions (range: 10.8–13.7%, *p* < 0.05). Another study by Thobois et al. (56) found no significant difference in execution time between MI and ME. Samuel et al. (57) demonstrated that when performing the task, patients with PD compared to HS in the MI group showed a decrease in activity in the dorsolateral and mesial frontal cortex (*p* < 0.01), whereas in the ME group, there was a decrease in the right dorsolateral frontal cortex and basal ganglia (*p* < 0.01). The ability to retain previously made movements in MI as well as in ME was not different between PD and HS groups (57).

#### 3.3.14 Main results for varied upper limb tasks (6 studies: 223 participants)

Yágüez et al. (39) conducted a pre-post-clinical trial with patients with PD. They examined the writing movement and execution time to perform ideograms. The intervention was first a practice phase in MI and then a phase in ME. A significant difference was observed in execution time between the baseline and post-ME practice sessions (*p* = 0.01) as well as between the post-MI and post-ME sessions, with an improvement after the ME practice phase (*p* = 0.03).

Sabaté et al. (40) demonstrated that sequential finger movements took 70% (*p* < 0.001) longer in MI and 80% (*p* < 0.001) longer in ME for patients with PD when compared to HS. Regarding the difference between MI and ME in patients with PD, Sabaté et al. (58) found a significant difference in favor of ME in execution time for a fast cyclic (*p* < 0.001) and a slow continuous movement task (*p* < 0.001), but no significant difference was found for a slow cyclic movement task. Bek et al. (59) demonstrated that action observation influences hand movement amplitude in PD patients, and MI increases the effects of action observation in these patients. People with PD may benefit from interventions that combine action observation with MI.

Gündüz and Kiziltan (60) analyzed F-waves during thumb abduction. They found that the average amplitude of F-waves significantly increased during MI and ME compared to rest conditions in both patients with PD non-apraxia (*p* < 0.001) and HS (*p* = 0.01) groups. Tremblay et al. (61) measured the MEP amplitude of two hand muscles both during the resting state and during the MI of a scissors-cutting task. No significant change was detected between conditions in patients with PD, while a significant difference was found in patients with HS (*p* < 0.05).

#### 3.3.15 Main results for verbal task (1 study: 10 participants)

Péran et al. (64) compared three tasks in patients with PD: *object naming*, an *action word* related to the object, and a *mental simulation* of the action with the object. They found that in contrast to object naming, mental simulation demonstrated a greater degree of activation in the prefrontal cortex bilaterally and in the parietal-occipital junction bilaterally (*p* < 0.001).

#### 3.3.16 Main results for the laterality judgment task (5 studies: 228 participants)

The task of lateral judgment involves an implicit MI process. Four studies (50, 72–74) divided the participants into groups based on their most affected side. Amick et al. (72) found that patients in the PD right-sided symptoms group made more errors than the HS in judging laterality (*p* = 0.01), but the left-sided symptoms group did not show a significant difference in error rates compared to the HS group. The results of Conson et al. (73) showed that patients with PD had a greater reaction time to determine the laterality of a body that corresponded to their most affected side compared to the other side (range: *p* < 0.01–0.03). However, no significant difference was found in terms of reaction time or accuracy between patients with right-sided symptoms and patients with left-sided symptoms (73). In the Dominey et al. (50) study, patients with PD were slower than patients with HS in determining letter symmetry and hand laterality (*p* < 0.001). Scarpina et al. (74) and Helmich et al. (67) conducted a similar protocol and found no significant differences in reaction time and accuracy among patients with PD with right-sided symptoms and HS, patients with PD with left-sided symptoms and HS, and between patients with PD with and without tremor and HS. Additionally, patients with PD with tremors demonstrated higher levels of imagery-related activity in the somatosensory area 3a when compared to both patients with PD without tremors and HS (*p* < 0.01) (67).

#### 3.3.17 Main results for MI tests and questionnaire (6 studies: 252 participants)

Heremans et al. (29, 68) used an adapted version of the BBT, consisting of wooden blocks measuring 2.5 cm^2^ and a box that was divided into 2 equal partitions measuring 18-cm high. Participants were instructed to transport 20 blocks as fast as possible from one side of the box to the other. This task was performed under four conditions: (a) ME, (b) MI with visual cues, (c) MI with auditory cues, and (d) MI without cues. Each condition was repeated three times in a random order. During execution, the box was placed at the participants’ midline, with the compartment holding the blocks pointing toward the hand being tested. During MI with visual cues, free vision of the box and blocks was provided. During MI with auditory cues, the box was removed from the participant’s sight. Instead, auditory cues were provided by a metronome at a rate of 0.5 Hz, and the participants were instructed to align every tic with the imagined pick-up of one block. During MI without cues, no visual or auditory information was provided. They found that patients with PD were slower on the BBT in MI and ME compared to HS (range: 16.7–30.4%; *p* < 0.01–0.02). Regarding the impact of cues in BBT, wherein the time required to transport 20 blocks was assessed using a mental chronometry paradigm, the execution time revealed no significant difference between MI with cues and ME. However, MI without cues was significantly slower than ME (*p* < 0.05).

Several studies used MI tests and questionnaires. There was no significant disparity observed between patients with PD and HS for the MIQ-R, KVIQ-20, Chaotic Motor Imagery Assessment (CMIA), and GIQ. Heremans et al. (68) and Peterson et al. (75) investigated KVIQ in patients with PD phase *on*, *off*, and HS, and no significant difference was found among groups. For the GIQ, no significant distinction was found between patients with PD with FOG and without FOG (73).

Kobelt et al. (41) used the short version of the KVIQ (KVIQ-10), which contains 10 items. There are three subscales: KVIQ visual (5–25), KVIQ kinesthetic (5–25), and KVIQ kinesthetic + visual (10–50). The scales are defined as both visual and a kinesthetic 5-point Likert scales ranging from 1 to 5 (1 = “no image”/“no sensation,” 5 = “image as clear as seeing it”/“as intense as moving”). The mean scores of the subscales were calculated. The five participating PD patients scored an average of 3.3 points higher on the visual subscale of the KVIQ-10 than on the kinesthetic subscale (41).

In order to evaluate MI perspectives in patients, Gäumann et al. (42) used two photographs of each item of the KVIQ: one photograph representing the internal perspective and one representing the external perspective. After each KVIQ item, patients were asked to identify which photograph represented their preferred perspective. Among patients with PD, 71.5% preferred an internal perspective (a first-person view), 26.3% chose an external perspective (a third-person view), 0.4% selected both perspectives, and 2.3% were unable to choose a perspective. When assessed with the KVIQ kinesthetic subscale, which measures the intensity of sensations, 73.3% of patients with PD preferred an internal perspective, 25.2% preferred an external perspective, 0.3% preferred both perspectives, and 1.4% did not select any perspective.

In the study conducted by Bek et al. (59), no significant differences were observed between the two groups on either the visual or kinesthetic subscales of the KVIQ. Additionally, task-specific ratings of visual and kinesthetic imagery were similar between the groups both before and after MI instructions (see Table 3). Both groups, however, exhibited a significant increase in the use of kinesthetic imagery (PD: *Z* = 2.73, *p* = 0.01; control: *Z* = 3.47, *p* < 0.001) and visual imagery (PD: *Z* = 2.45, *p* = 0.01; control: *Z* = 3.15, *p* < 0.001) following MI instructions. The control group also reported enhanced vividness of sensations (*Z* = 2.14, *p* = 0.03) and images (*Z* = 2.35, *p* = 0.02) after instructions, whereas the PD group exhibited no significant alteration in the vividness of either sensations or images.

#### 3.3.18 Main results for neurofeedback intervention (2 studies: 28 participants)

Tinaz et al. (69) found that the intensity and quality of body sensations evoked during MI and the emotional and motivational context of MI determined the direction (i.e., negative or positive) of the insula-dorsomedial frontal cortex’s functional connectivity. After 10–12 neurofeedback sessions with successful MI strategies, all subjects showed a significant increase in the insula-dorsomedial frontal cortex’s functional connectivity. The MI strategies encompassed movements associated with diverse activities and exercise routines, such as walking, running, lifting weights, and swimming. There was no significant difference in patients with PD between pre-and post-intervention on the MDS-UDPRS-III score. Subramanian et al. (70) demonstrated in a study involving PD patients an early stage of the disease. Out of 10 participants, 5 were in the experimental group (with feedback), and the remaining 5 were in the control group (without feedback). There was a significant improvement of 37% (*p* = 0.04) in the UPDRS score between pre-and post-intervention in the experimental group, whereas the control group showed no significant difference.

#### 3.3.19 Main results for MI of the whole body (1 study: 22 participants)

Mori et al. (71) measured rCBF in patients with PD and HS while in a standing position. During MI, no significant difference was shown between groups. During ME, patients with PD against HS exhibited a significant increase in the right cerebellar vermis and left paracentral gyrus and a significant decrease in the bilateral middle frontal gyrus.

## 4 Discussion

Since the 1980s, motor imagery has been used in sport and performance activities and has attracted considerable interest (76). This technique has been adapted to PD patients’ rehabilitation with promising results, despite the limited number of RCT studies published (22–25, 31–38). Among the 53 included studies, there were few RCTs (12 studies) with an average PEDro score of 6.6, which can be considered as medium to high quality. The protocol and outcomes measured were heterogeneous, and there were no RCTs with specific outcomes for upper limbs or speech other than the UPDRS score. The population of RCTs and descriptive studies was relatively young with a low severity level (i.e., H&Y score). In fact, most RCTs excluded patients with scores greater than 3. Therefore, it is not possible to conclude the applicability of MI in patients with PD who have a higher severity. Hence, MI should be used as early as possible before cognitive impairment prevents its use. Taking these aspects into account, the results should be treated with caution, as methodological biases must be resolved before conclusions can be drawn.

In addition to RCTs, we also investigated descriptive and non-RCTs to determine how MI has been used in the PD population. It is also found that patients with PD have similar scores to HS in MI questionnaires (such as KVIQ, MIQ-R, and GIQ), which means that they can practice MI. The presence of cues (visual and auditory) was also found to improve the abilities of patients with PD in MI.

The MI of walking can be employed along a corridor of different lengths, using the time taken for execution as a method of measurement. Walking speed and TUG can be interesting outcomes to be assessed at regular intervals to monitor progress.

Motor symptoms assessed by the UPDRS showed no significant difference between the two groups (intervention vs. control) in the RCTs. However, Part 3 of the UPDRS comprises items for both the upper and lower limbs, and it has been observed that the RCTs were specifically directed toward the lower limbs. As the MI protocol did not encompass all aspects evaluated in the UPDRS, this may explain why there was no change (77).

Even though we did not establish date limits, we were unable to include many studies. Indeed, this is a recent topic of interest, as the initial study included herein was published in 1997, while the initial RCT included in this review dates from 2007. Among the studies that were excluded, there were 21 ongoing clinical trials whose results have not yet been fully published. Additional details regarding these studies are expected to be made available in the near future. This study aimed to guide and facilitate the use of MI in clinical practice, as well as to highlight the main results observed in these studies in terms of improvements in motor symptoms, balance, gait, and quality of life. Indeed, MI is a technique that does not necessitate any equipment, is easy and safe to set up, and merely requires a learning phase beforehand. In a context where the prevalence of PD is increasing, it is important to empower patients and provide them with tools they can use at home to complete other treatments.

The main limitation of this study was the fact that, in descriptive and non-RCT studies, only the main tasks and outcomes of MI were analyzed. Our primary emphasis was on the tasks and outcomes most commonly used in MI-related clinical research. However, there may be other fascinating areas that remain unexplored, such as activities that involve the dual-task paradigm, where motor and cognitive tasks are performed simultaneously. Additionally, a noteworthy limitation of this review is that the most significant studies, particularly RCTs, did not include patients with the most severe forms of PD. Consequently, it remains unclear whether the recommendations provided here apply to individuals with more advanced stages of the disease.

Despite the limited number of RCTs focusing on MI in patients with PD, combined with diverse protocols, outcomes, and potential biases, the findings offer a promising outlook, particularly in addressing walking and balance impairments. However, research into upper limb function or speech remains scarce. Future studies in this field must involve larger cohorts of participants and adopt more precise protocols tailored to the unique challenges posed by upper limb impairments. The criteria for assessing outcomes related to walking and balance align with recommendations from the French National Authority for Health, which provides a valuable standard for evaluating MI interventions in PD.

In conclusion, it is imperative to acknowledge that this scoping review underscores the necessity for further research and revisions in the forthcoming years. The ongoing RCTs registered in clinical trial databases highlight the evolving landscape of MI interventions for PD, suggesting that a comprehensive and updated systematic review will be essential to capture the latest advancements and insights in this field.

## Data Availability

The original contributions presented in the study are included in the article/supplementary material, further inquiries can be directed to the corresponding author/s.
